# Periosteum contains skeletal stem cells with high bone regenerative potential controlled by Periostin

**DOI:** 10.1038/s41467-018-03124-z

**Published:** 2018-02-22

**Authors:** Oriane Duchamp de Lageneste, Anaïs Julien, Rana Abou-Khalil, Giulia Frangi, Caroline Carvalho, Nicolas Cagnard, Corinne Cordier, Simon J. Conway, Céline Colnot

**Affiliations:** 1 INSERM UMR1163, Imagine Institute, Paris Descartes University, 75015 Paris, France; 2 Paris-Descartes Bioinformatics Platform, 75015 Paris, France; 3 INSERM US24 - CNRS UMS3633 Cytometry Platform, Paris Descartes University, 75015 Paris, France; 4 Herman B. Wells Center for Pediatric Research, Department of Pediatrics, Indiana University School of Medicine, Indianapolis, IN 46202 USA

## Abstract

Bone regeneration relies on the activation of skeletal stem cells (SSCs) that still remain poorly characterized. Here, we show that periosteum contains SSCs with high bone regenerative potential compared to bone marrow stromal cells/skeletal stem cells (BMSCs) in mice. Although periosteal cells (PCs) and BMSCs are derived from a common embryonic mesenchymal lineage, postnatally PCs exhibit greater clonogenicity, growth and differentiation capacity than BMSCs. During bone repair, PCs can efficiently contribute to cartilage and bone, and integrate long-term after transplantation. Molecular profiling uncovers genes encoding Periostin and other extracellular matrix molecules associated with the enhanced response to injury of PCs. *Periostin* gene deletion impairs PC functions and fracture consolidation. Periostin-deficient periosteum cannot reconstitute a pool of PCs after injury demonstrating the presence of SSCs within periosteum and the requirement of Periostin in maintaining this pool. Overall our results highlight the importance of analyzing periosteum and PCs to understand bone phenotypes.

## Abstract (web summary)

The periosteum, a tissue lining the bone surface, and the bone marrow are known to contain bone-forming cells. Here the authors show that skeletal stem cells reside in the mouse periosteum, and that periosteal cells have common embryonic origins with bone marrow stromal/stem cells (BMSCs), but are better at bone repair and long-term integration than BMSCs.

## Introduction

The skeleton is a central component of vertebrates’ body, providing structural support and protection for major organs. The 206 bones constituting the human skeleton store vital minerals, form muscle attachments, and comprise the niche for hematopoiesis. Bones are constantly challenged mechanically and can remodel or regenerate throughout life. The development, growth, and regeneration of this essential organ system rely on two robust ossification processes, intramembranous ossification occurring by direct differentiation of mesenchymal precursors into osteoblasts and endochondral ossification marked by the formation of an intermediate cartilage template^1^. Vascular invasion of this cartilage template drives the replacement of cartilage by the bone marrow cavity and bone. During this crucial step of skeletal development, hematopoietic stem cells (HSCs) migrate into the developing bone to establish their niche within the marrow cavity. In parallel, bone-forming cells distribute in various bone compartments along the inner surface of bone (endosteum), metaphyseal trabeculae, and on the outer surface of the bone within the periosteum. It is well established that these two processes of ossification can be recapitulated postnatally to very efficiently repair injured bones^2–5^. This reactivation of the skeletogenic program requires the re-expression of key transcription factors and growth factors regulating skeletal development. Yet the skeletal stem cells (SSCs) that permit this regenerative process and the mechanisms of stem cell activation in response to bone injury remain elusive.

Research on the biology of SSCs has mostly concentrated until now on the characterization of bone marrow stromal cells/skeletal stem cells (BMSCs), that form the niche for HSCs, regulate bone turnover, and show multipotency and self-renewal capacities after subcutaneous transplantation^6–11^. SSC populations are very heterogeneous, making it a challenge to identify specific markers to trace these cells in vivo. Recent advances with genetic mouse models have identified several markers to define various sub-populations of SSCs that appear during limb development and post-natal growth, and play a role in bone maintenance and repair^12–22^. However, these markers do not distinguish the tissue origins of activated SSCs in response to bone injury. Although BMSCs are largely used for enhancing bone repair through cell-based therapy, it has become clear that BMSCs are not the central cellular component of endogenous skeletal repair. In contrast, the periosteum is largely involved in bone strength maintenance and its preservation is crucial for normal bone repair^23–31^. The periosteum is a thin layer of vascularized tissue lining the bone surface, supporting the tendon and muscle attachments, and highly responsive to mechanical stress. Several studies have revealed the periosteum as a major source of SSCs for bone repair, but this population has been largely overlooked until now^30,32,33^. We hypothesized that bone marrow and periosteum comprise SSC populations with distinct functions in bone biology and specifically during endogenous bone repair.

Here we uncover common embryonic origins of BMSCs and periosteal cells (PCs), but increased regenerative capacities and long-term integration of PCs during bone regeneration in mice. Periosteum grafting shows that a pool of PCs is reconstituted and maintained within periosteum in response to injury and can be re-activated after subsequent injuries revealing the presence of SSCs within periosteum. Molecular profiling of PCs and BMSCs in response to injury identifies specific factors expressed in the extracellular matrix (ECM) of periosteum, including Periostin. Bone repair is compromised in *Periostin* KO mice due to impaired periosteum and PC functions. Unlike wild-type periosteum, Periostin-deficient periosteum cannot reconstitute a pool of PCs and contribute to healing after successive bone injuries causing severe repair defects. Periostin is, therefore, a key regulator of SSCs in periosteum and their niche.

## Results

### PCs and BMSCs share specific markers

**Fig. 1:**
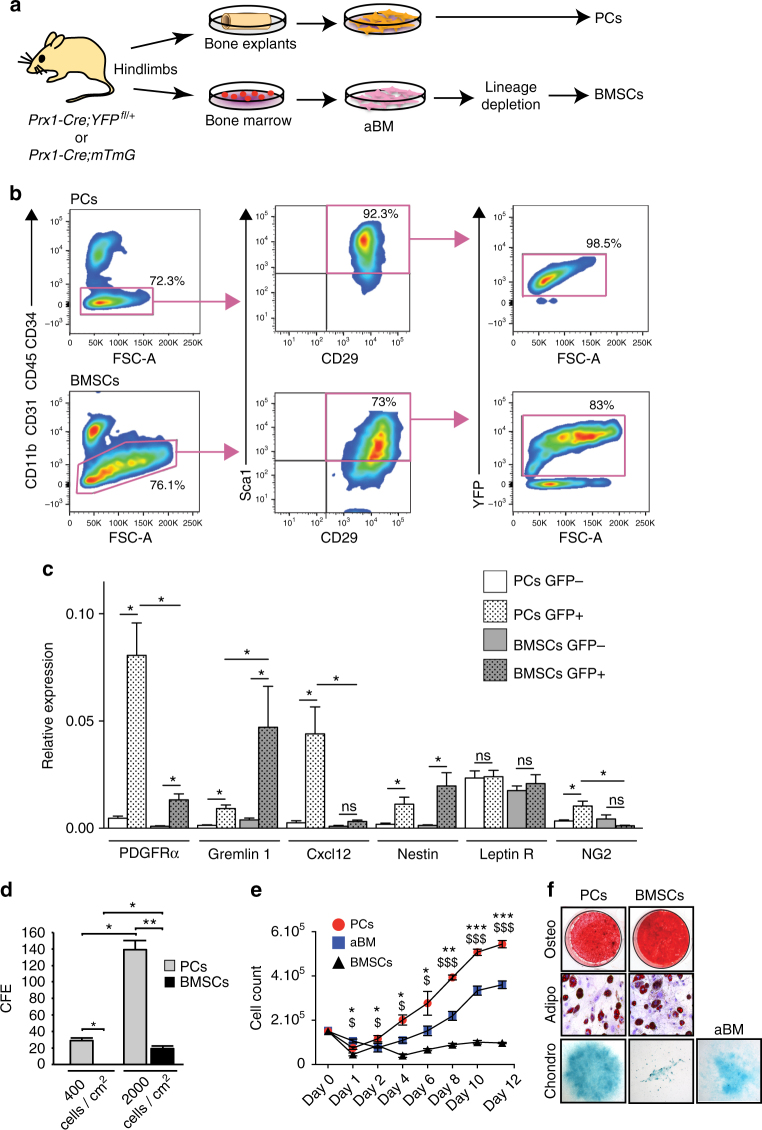
FACS and in vitro analyses of PCs and BMSCs. **a** Experimental design of periosteal cells (PCs) and bone marrow stromal cells/skeletal stem cells (BMSCs) cultures from *Prx1-Cre;YFP*^*fl/+*^ or *Prx1-Cre;mTmG* mouse hindlimbs. Bone marrow cells were flushed from hindlimbs and plated to obtain adherent bone marrow cells (aBM). After expansion, lineage depletion was performed to isolate BMSCs with no further passage. The flushed bones were placed in culture to isolate in one step the PCs migrating out of the explants. **b** Flow cytometry analyses of PCs and BMSCs isolated from *Prx1-Cre;YFP*^*fl/+*^ mice. PCs and BMSCs negative for endothelial/hematopoietic markers (CD31, CD11b, CD34, and CD45) and double-positive for Sca1/CD29 are largely YFP+ (derived from Prx1-mesenchymal lineage). **c** Quantitative RT-PCR analyses of FACS sorted GFP-positive and GFP-negative PCs and BMSCs isolated from *Prx1-Cre;mTmG* mice. Results show overexpression of the markers *PDGFRα*, *Gremlin1*, *Cxcl12*, and *Nestin* and to a lesser extent *NG2* in GFP-positive compared to GFP-negative PCs, but not *LeptinR*. **d** CFE assays showing PCs forming colonies at cell density as low as 400 cells/cm^2^ 14 days after plating and BMSCs at 2000 cells/cm^2^ 14 days after plating. Colonies were stained with Giemsa blue and counted under microscope. **e** Cell-growth assay shows that PCs grow faster than adherent bone marrow cells (aBM) and BMSCs. The cells were plated at the same density (10^5^ cells/dish) and counted every day during the first two days then every two days for 12 days (* represents the comparison between PCs and aBM, $ represents the comparison between PCs and BMSCs). **f** In vitro differentiation of PCs and BMSCs into osteogenic (3 weeks), adipogenic (3 weeks), and chondrogenic (2 weeks) lineages as shown by alizarin red S, Oil red O, and alcian blue staining, respectively. Due to the poor chondrogenic capacity of BMSCs, aBM were assessed for chondrogenesis. Statistical differences between the groups (*n* = 3 or 4 per group) were determined using Mann–Whitney test (*,$ p ≤ 0.05, **,$$ p < 0.001, ***,$$$ p < 0.0005). All data represent mean ± SD

In the absence of a unique marker to define SSCs, we used Prx1, a marker of the mesenchymal lineage in developing limbs^34,35^. BMSCs were obtained by flushing bone marrow of tibias and femurs followed by lineage depletion. Remaining long bones free of bone marrow were placed in culture and PCs were let to grow out of the bone explants (Fig. 1a and Supplementary Fig. 1a). In primary cultures of PCs and BMSCs isolated from *Prx1-Cre;YFP*^*fl/+*^ mice, the populations negative for hematopoietic and endothelial makers and double-positive for Sca1/CD29 and Sca1/CD105^36^ were mostly Prx1-derived YFP-positive (Fig. 1a–b and Supplementary Fig. 1b). The populations that were positive for hematopoietic and endothelial makers were mainly YFP-negative (Supplementary Fig. 1c). By qRT-PCR, Prx1-sorted PCs from *Prx1-Cre;mTmG* mice overexpressed markers previously shown to define mouse BMSCs, such as *PDGFRα*^37^, *Gremlin 1*^19^, *Cxcl12*^8^, *Nestin*^15^, but not *Leptin Receptor* (*Leptin R*)^20,21^. Prx1-sorted PCs also overexpressed the pericyte marker *NG2*^38^ and did not overexpress the fibroblast marker *Vimentin* compared to the Prx1-negative population^39^ (Fig. 1c and Supplementary Fig. 1d). Secondary colony forming efficiency assay (CFE) show higher clonogenicity of PCs compared to BMSCs (Fig. 1d). Cell-growth analyses revealed higher cell growth of PCs compared with adherent bone marrow cells (aBM, prior to lineage depletion) and BMSCs (Fig. 1e). PCs can differentiate in osteogenic, adipogenic, and chondrogenic lineages in vitro with an increased potential for chondrogenesis compared to BMSCs and aBM (Fig. 1f).

### Common embryonic origin of PCs and BMSCs

**Fig. 2:**
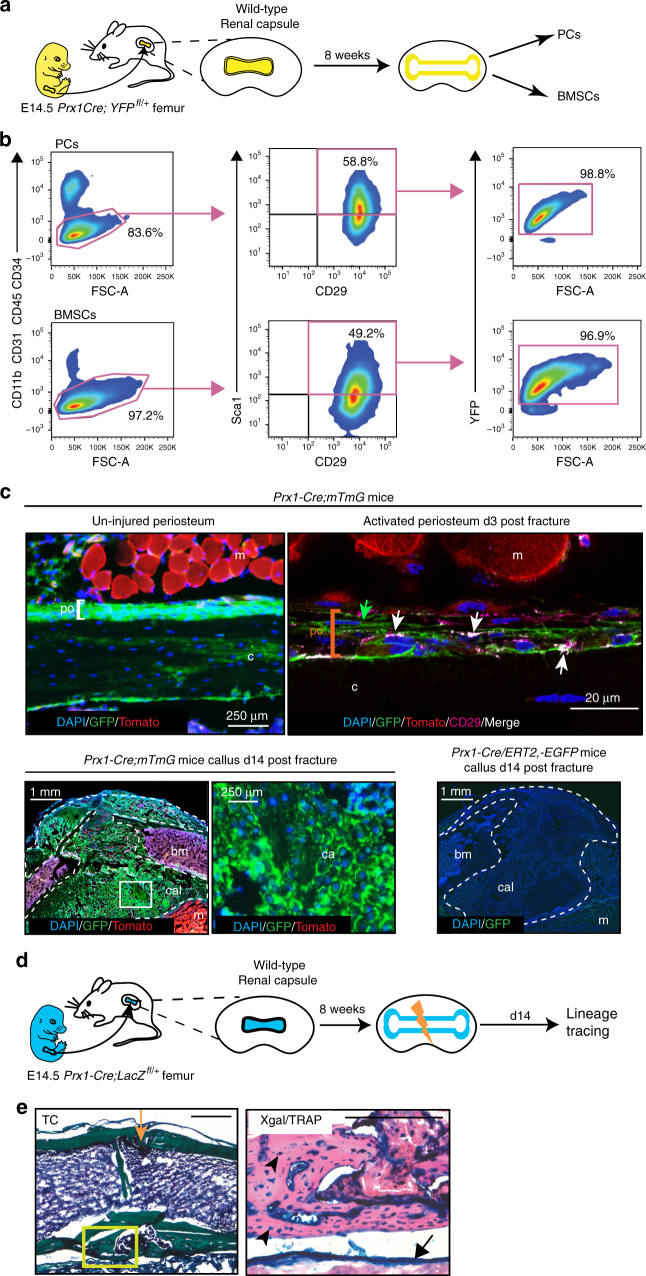
PCs and BMSCs derive from the Prx1-mesenchymal lineage. **a** Experimental design for renal capsule transplantations. Femoral cartilages before vascular invasion were isolated from E14.5 *Prx1-Cre;YFP*^*fl/+*^ embryos and transplanted under the renal capsule of wild-type hosts. PCs and BMSCs were isolated from mature skeletal elements 8 weeks post-transplantation as shown in Fig. 1a. **b** Flow cytometry analyses of PCs and BMSCs isolated from *Prx1-Cre;YFP*^*fl/+*^ mature skeletal elements grown under renal capsule. Both PCs and BMSCs that are negative for endothelial/hematopoietic markers (CD31, CD11b, CD34, and CD45) and positive for Sca1/CD29 are mostly YFP-positive (Prx1-donor-derived) (*n* = 3 per group). **c** Localization of Prx1-derived cells in the periosteum and fracture callus of *Prx1-Cre;mTmG* mouse. The un-injured periosteum is derived from Prx1-lineage (GFP+). In the activated periosteum at day 3 post fracture, some Prx1-derived cells (GFP/pointed by green arrows) colocalize with CD29-positive cells (Merge CD29+ GFP+/pointed by white arrows). In the fracture callus (d14), all chondrocytes and osteoblasts/osteocytes are derived from Prx1-lineage (GFP+). The fractures performed on *Prx1-Cre/ERT2*,*-EGFP* mice, where EGFP is expressed under the Prx1 promoter, show no GFP signal in the callus. **d** Experimental design for cell-lineage analyses of Prx1-derived cells during bone regeneration in renal capsule. Femoral cartilages were isolated from E14.5 *Prx1-Cre*^*+/−*^*;LacZ*^*fl/+*^ embryos and transplanted under the renal capsule of wild-type hosts. After 8 weeks, mature femurs underwent osteotomy and were collected at d14 post-fracture for cell-lineage tracing. **e** TC and Xgal/TRAP double-staining on longitudinal sections of *Prx1-Cre*^*+/−*^*;LacZ*^*fl/+*^ fractured femurs in wild-type hosts (top) showing new bone within the callus entirely donor-derived, i.e., LacZ + TRAP− (black arrowheads: osteocytes) and some osteoclasts (TRAP+ LacZ+ with endogenous beta-galactosidase activity). Scale bar: 0.5 mm. TC: Masson’s trichrome, TRAP: Tartrate resistant acid phosphatase, m: muscle, c: cortex, po: periosteum, bm: bone marrow, cal: callus, ca: cartilage, white dashed line: callus, orange lightning bolt: fracture, orange arrow: fracture site, black arrow: points to the periosteum, black arrowhead: osteocytes in new bone

BMSCs form the niche for HSCs and are established within the bone marrow compartment when cartilage anlagen are vascularized during long bone development^40–42^. In mice, this step occurs from the developmental stage E14.5 during the formation of the primary ossification center. Simultaneously, the periosteum forms and will include PCs. Whether BMSCs and PCs derive from the same pool of mesenchymal cells within each skeletal element or whether BMSCs can be brought by blood vessels from another local or systemic source is still not well understood. To address this question, we performed renal capsule transplantations for cell-lineage analyses. On one hand we tracked cells derived from transplanted cartilage elements that are not yet vascularized and do not yet comprise a bone marrow compartment. On the other hand, we tracked cells brought by the wild-type host vasculature that support the vascularization of cartilage grafts in the renal capsule^43,44^ (Fig. 2a). First we transplanted E14.5 *Prx1-Cre;YFP*^*fl/+*^ grafts into wild-type hosts. In fully developed bones 8 weeks post-transplantation, the PC and BMSC populations positive for Sca1/CD29/CD105 coincided with the donor-derived YFP-positive population marked by Prx1 (Fig. 2b and Supplementary Fig. 2a). Therefore, PCs and BMSCs were both derived from the transplanted cartilage element. Conversely, after transplantation of E14.5 wild-type femoral cartilages into *Prx1-Cre;YFP*^*fl/+*^ hosts, PC and BMSC populations positive for Sca1/CD29 were YFP-negative, confirming that PCs and BMSCs are derived from the graft and not from the host. These results show that both BMSCs and PCs are derived from the local Prx1-mesenchymal lineage forming each embryonic skeletal element and are not brought by blood vessels during the establishment of the primary ossification center (Supplementary Fig. 2b, c).

### Local recruitment of Prx1-derived cells during bone repair

The Prx1-derived cells within adult bones have been shown to participate in bone repair^34,45^. We localized Prx1-derived cells within the intact periosteum and activated periosteum 3 days post-fracture in adult *Prx1-Cre;mTmG* mice in which Prx1-derived cells are GFP-positive and all other cells are Tomato-positive (Fig. 2c). In the activated periosteum, we detected Prx1-derived cells also marked by CD29 (Fig. 2c, Merge). By day 14 post-fracture, all cells contributing to cartilage and bone in the callus were GFP-positive Prx1-derived, indicating that Prx1 marks all stem/progenitor cells recruited to form the fracture callus (Fig. 2c). To verify that the signal was due to Cre recombination during development and not in response to injury, we performed fractures in *Prx1-Cre/ERT2-EGFP* mice that express EGFP under the Prx1 promoter, and observed no GFP signal in the callus (Fig. 2c). To distinguish the systemic vs. local recruitment of cells in the callus, we transplanted E14.5 *Prx1-Cre;LacZ*^*fl/+*^ femoral cartilage grafts into wild-type renal capsules. Eight weeks post transplantation, the fractures were performed on the fully developed bones derived from the grafts and lineage analyses showed that all bone cells within the fracture callus at d14 were LacZ-positive donor-derived (Fig. 2d, e). Controls showed absence of LacZ-positive host-derived osteoblasts/osteocytes in the callus, when wild type E14.5 femoral grafts were transplanted in *Prx1-Cre;LacZ*^*fl/+*^ hosts (Supplementary Fig. 2d, e). Although several studies have suggested a potential systemic recruitment of SSCs for bone repair^46^, these results show that this systemic recruitment does not occur for endogenous bone repair and that the Prx1-derived cells forming the fracture callus are all recruited locally.

### Higher regenerative capacity of PCs compared to BMSCs

**Fig. 3:**
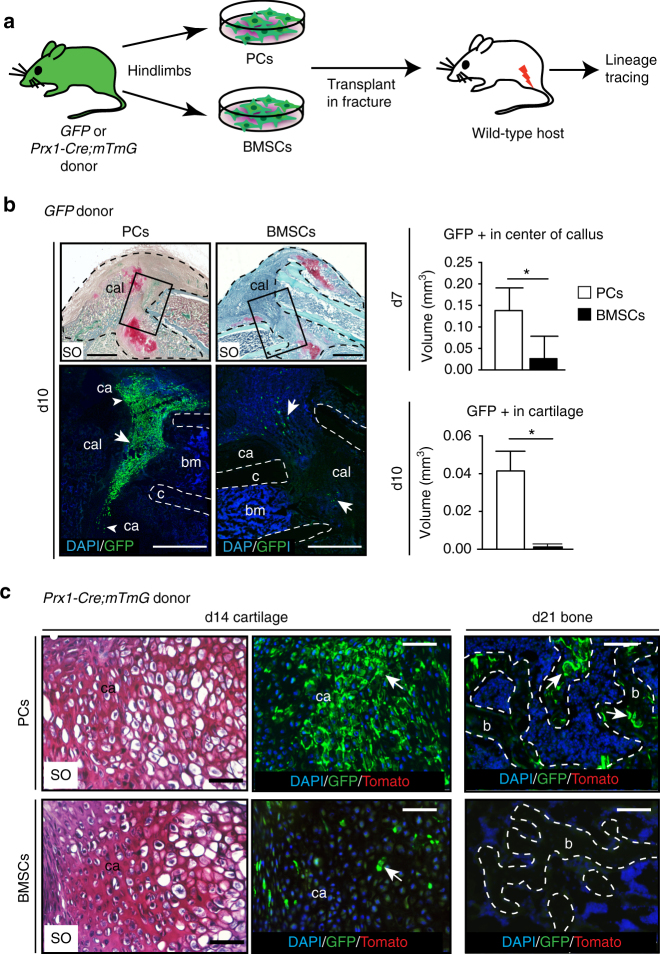
PCs integrate efficiently into the fracture callus. **a** Experimental design for the isolation of PCs and BMSCs from hindlimbs of *GFP* or *Prx1-Cre*;*mTmG* donor mice and transplantation at the fracture site of wild-type hosts. **b** Lineage tracing of GFP + cells in the fracture callus. SO staining and DAPI/GFP immunofluorescence on longitudinal sections of mouse fractured tibias at day 10 post-transplantation shows PCs migrating very far in the callus (white arrow) and integrating in cartilage (white arrowhead). Histomorphometric analyses of the volume occupied by GFP + cells showing increased volume for PCs compared to BMSCs in the center of the callus at d7 (*n* = 5 per group) and increased volume in cartilage by day 10 (d10) (*n* = 4 per group). Black dashed line: callus, white dashed line: bone cortex, white arrows point to transplanted cells. Scale bar: 1 mm. **c** SO staining and DAPI/GFP/Tomato signals on longitudinal sections of wild-type mouse fractured tibias at days 14 (d14) and 21 (d21) post-transplantation of PCs (left column) or BMSCs (right column) isolated from *Prx1-Cre;mTmG* donors. High magnification of SO staining showing hypertrophic cartilage in the center of the callus and DAPI/GFP/Tomato signals on adjacent sections showing PCs and BMSCs Prx1-derived chondrocytes only marked by GFP (and Tomato-negative) at d14 (white arrows). By d21, PC Prx1-derived osteocytes marked by GFP (white arrows) were found in new bone (delimited by white dashed line), but no BMSC Prx1-derived osteocytes were detected. Scale bar: 125μm. SO: Safranin-O/Fast Green, cal: callus, c: cortex, ca: cartilage, b: bone. Statistical differences between the groups were determined using Mann–Whitney test (*p ≤ 0.05, **p < 0.001). All data represent mean ± SD

To directly compare the regenerative potential of PCs and BMSCs to bone repair in vivo, we transplanted GFP-labeled PCs and BMSCs in the fracture site of wild-type adult mice for in vivo lineage tracing (Fig. 3a). By day 7-post fracture (d7), transplanted PCs were found in the center of the callus and showed increased contribution to the callus compared to BMSCs (Fig. 3b and Supplementary Fig. 3a). Similar results were obtained for aBM and BMSC populations indicating that cell depletion of bone marrow cells did not compromise their biological activity (Supplementary Fig. 3a). This increased contribution of PCs was not due to changes in cell proliferation or cell death compared with BMSCs (Supplementary Fig. 3b and c). The majority of BMSCs stayed at the periphery of the callus and PCs integrated far into the callus and cartilage by day 10 (Fig. 3b and Supplementary Fig. 3b, BMSCs right panel). Wound healing assays showed that PCs migrated faster than BMSCs in vitro, which could at least in part explain their higher regenerative potential in vivo (Supplementary Fig. 3d). Lineage analyses of PCs and BMSCs isolated from *Prx1-Cre;mTmG* donors showed that PCs derived exclusively from the Prx1-mesenchymal lineage contributed to cartilage and bone within the callus, while BMSCs had less potential to form cartilage and did not participate in forming new bone at later stages (Fig. 3c).

### Molecular profiling of the periosteum response to injury

**Fig. 4:**
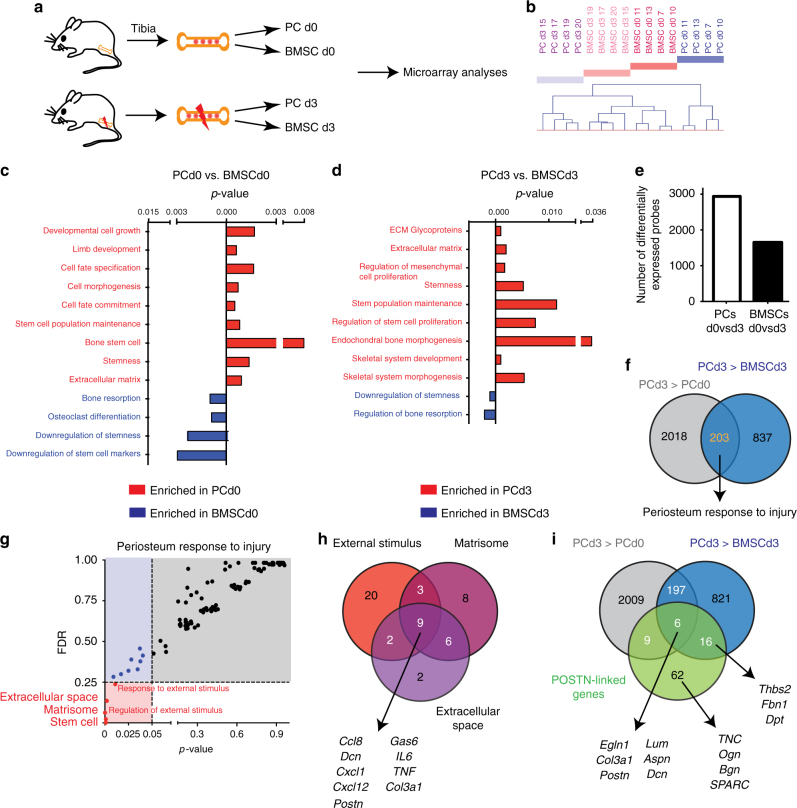
Microarray analyses of PCs and BMSCs in response to fracture. **a** Experimental design for microarray analyses of PCs and BMSCs isolated from wild-type un-injured tibias (d0) and from tibias 3 days post fracture (d3) (*n* = 4 per group). **b** Hierarchical clustering of biological replicates. **c**, **d** GSEA analyses of PCd0 vs. BMSCd0 and PCd3 vs. BMSCd3, respectively. PCs are enriched in stem cell, developmental, skeletal, and extracellular matrix gene sets (red) compared to BMSCs at both d0 and d3 (blue). **e** Number of differentially expressed probes in PCs and BMSCs in response to fracture. **f** Venn diagram showing the intersection of PCd3 vs. PCd0 and PCd3 vs. BMSCd3 representing the periosteum response to injury (PRI). **g** GSEA analysis of PRI genes. Red, blue, and gray boxes correspond to significant, interesting, and non-useful functions, respectively. Five significant functions are identified “response to external stimulus, “regulation of external stimulus”, “extracellular space”, “matrisome”, and “stem cell” (red). **h** The GSEA significantly enriched GO categories “response to external stimulus” and “regulation of external stimulus” were merged into “external stimulus” and compared by Venn to the “extracellular space” and “matrisome” GO categories resulting in a list of 9 common genes. **i** Venn diagram shows the intersection of PRI and *Postn*-linked genes resulting in a list of 6 genes (Complete list of 93 *Postn* linked genes in Supplementary Table 3)

In order to uncover the molecular signatures of PCs defining their high regenerative potential compared to BMSCs, we performed microarray analyses of PCs and BMSCs isolated from un-injured (d0) and injured (day 3 post-fracture) tibias (Fig. 4a). Sample clustering showed that all biological replicates clustered together and that PCs from un-injured bone (PCd0) are a distinct population compared to other groups in particular to BMSCd0. After fracture, PCd3 are closer to BMSCd3 (Fig. 4b). To identify gene sets that distinguish PCs and BMSCs before or after injury, we performed GSEA analyses comparing either PCd0 vs. BMSCd0 or PCd3 vs. BMSCd3. At both d0 and d3 post-fracture, PCs share common GO categories such as “stemness”, “limb development”, and “ECM”. In contrary, BMSCs are enriched in GO categories such as “downregulation of stemness”, “bone resorption”, and “immune and hematopoietic lineage” (Fig. 4c, d and Supplementary Fig. 4a, b). The number of differentially expressed genes in response to injury was greater in PCs compared to BMSCs (Fig. 4e). We then focused on genes specifically upregulated in PCs after fracture, but not in BMSCs and found 203 genes defining the “periosteum response to injury” (PRI) gene set (Fig. 4f). GSEA analysis comparing PCs and BMSCs showed that PRI genes are enriched in five different functions (Fig. 4g, in red). In order to find candidate genes that confer higher regenerative capacities to PCs, we excluded the “stemness” GO categories and merged “response to external stimulus” and “regulation of external stimulus” into “external stimulus”. We intersected the “external stimulus”, “matrisome”, and “extracellular space” gene sets to identify 9 candidate genes of interest (Fig. 4h). We focused on *Periostin* (*Postn*) gene previously described as being specifically expressed within periosteum^47^. Among the “*Postn*-linked genes” (complete list of *Postn*-linked genes in Supplementary Table 3), 6 genes were found in common with the PRI gene set and belong to the matricellular protein and small leucin rich proteoglycan families (Fig. 4i and Supplementary Table 3). Together, these findings reveal that PCs and BMSCs have distinct molecular profiles and that PCs are more responsive to bone injury. The molecular response to injury is marked in PCs with the early upregulation of genes encoding ECM proteins, which play important roles in cell–matrix interactions and may be key elements for SSC activation after bone injury.

### Lack of Periostin impairs periosteum function and bone healing

**Fig. 5:**
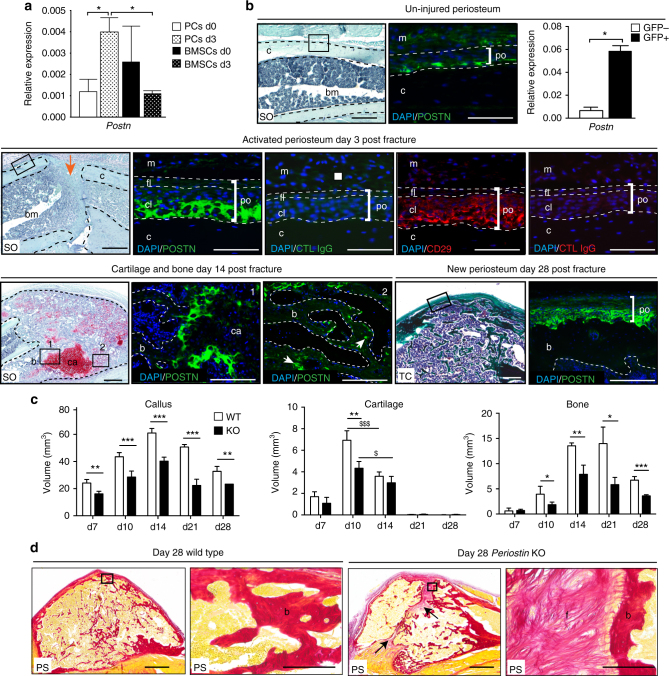
Periostin is required for adequate bone repair.** a** qRT-PCR analyses of PCs and BMSCs isolated from un-injured tibias and tibias 3 days post-injury. *Periostin* (*Postn)* is specifically upregulated in PCs in response to injury and in PCs compared to BMSCs in response to injury. **b** SO staining and DAPI/POSTN immunofluorescence on wild-type longitudinal tibia sections showing Periostin (POSTN) expressing cells in the un-injured periosteum near the cortex (immunofluorescence corresponds to box area in SO). qRT-PCR analyses show high *Postn* expression in Prx1-derived PCs (GFP+) sorted from PCs cultures of un-injured hindlimbs of *Prx1-Cre;mTmG* mice. Three days after fracture, POSTN is highly expressed in the cambial layer (cl) of the activated periosteum (GFP) coinciding with expression of CD29 (Red). At day 14 post-fracture, POSTN is expressed in hypertrophic cartilage at the junction between cartilage and bone within the callus (box 1, GFP) and in osteoblasts within new bone trabeculae (box 2, white arrows). By day 28, POSTN expression is high in the inner layer of the newly formed periosteum at the periphery of the remodeling callus. Scale bar: 0.5 mm. **c** Histomorphometric analyses of callus, cartilage, and bone volumes at days 7 (d7), 10 (d10), 14 (d14), 21 (d21), and 28 (d28) post fracture in wild type (WT) and *Periostin* KO (KO) mice. **d** Picrosirius red staining (PS) on longitudinal sections of fracture callus at d28 shows absence of consolidation and fibrosis in *Periostin* KO mice (black arrows). Scale bar: 1 mm. SO: Safranin-O/Fast Green, TC: Masson’s trichrome, m: muscle, c: cortex, po: periosteum, fl: fibrous layer, cl: cambial layer, b: bone, bm: bone marrow, ca: cartilage, f: fibrosis, CTL: non-immune IgG. Black dashed line: cortex (un-injured and day 3) or callus (day 14). White dashed line: periosteum (un-injured and day 3) or bone trabeculae (days 14 and 28). Statistical differences between the groups were determined using Mann–Whitney test (*p ≤ 0.05, **p < 0.001, ***p < 0.0005) (*n* = 3–5). All data represent mean ± SD

**Fig. 6:**
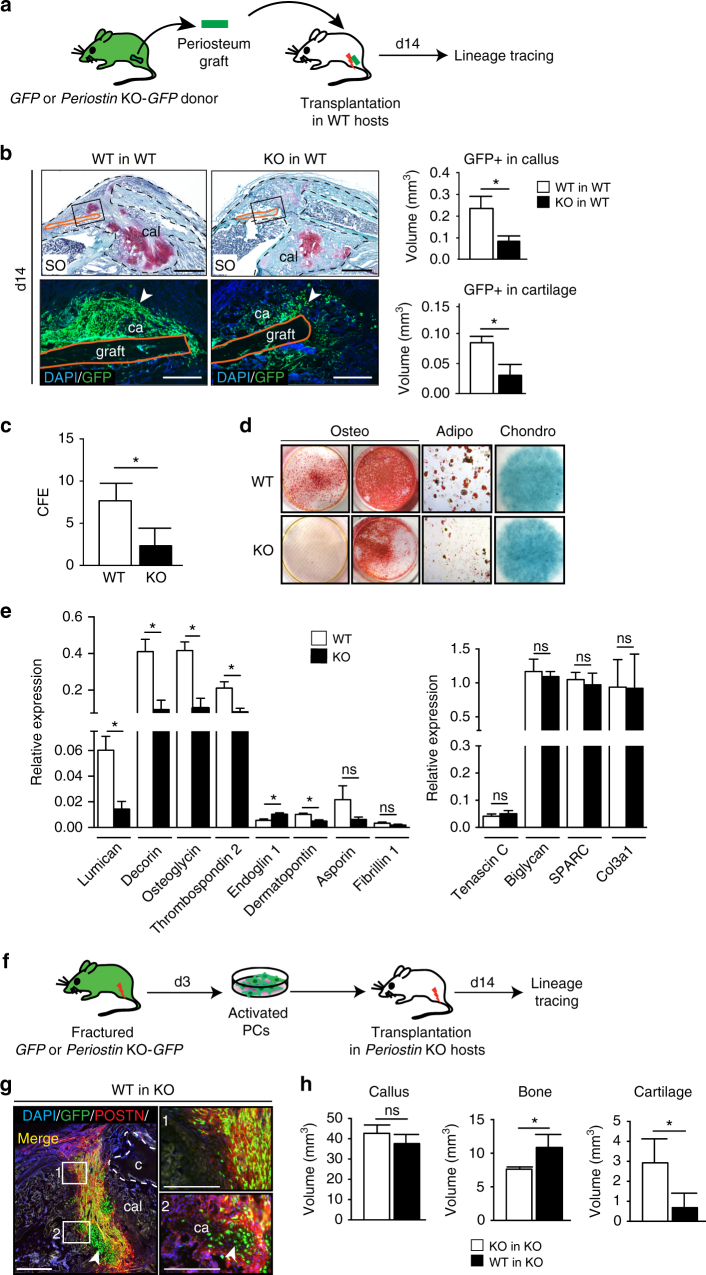
Impaired periosteum in *Periostin* KO mice. **a** Experimental design for the isolation of periosteum grafts from GFP or *Periostin* KO-GFP donors and transplantation at the fracture site of wild type (WT) hosts for lineage tracing of periosteum-derived cells during bone repair. **b** SO staining and DAPI/GFP immunofluorescence on longitudinal callus sections at day 14 reveals decreased contribution KO-GFP grafts (KO in WT) compared to GFP grafts (WT in WT) (arrowheads). Quantification of GFP signal shows decreased volume in callus and cartilage for KO-GFP grafts compared to GFP grafts. Scale bar: 1 mm. **c** CFE assay on activated PCs isolated from WT and KO mice and plated at 400 cells/cm^2^ for 14 days. Colonies were stained with Giemsa blue and counted. **d** In vitro differentiation assays of activated PCs isolated from WT and KO mice shows osteogenic differentiation (alizarin red S stain) at 2 weeks for WT PCs, but not for KO PCs (left) and at 5 weeks for WT and KO PCs (right). Adipogenesis (Oil red O) is reduced in KO at 3 weeks and chondrogenesis (alcian blue stain) at 1 week is similar in WT and KO PCs. **e** Quantitative RT-PCR analyses of *Periostin (Postn)*-linked genes, some of them upregulated in PCs in response to injury and encoding ECM proteins (see Fig. 4i and Supplementary Table 3) in *WT*-PCs and KO PCs. **f** Experimental design for the isolation of activated PCs from GFP or KO-GFP mice and transplantation at the fracture site of KO hosts (WT in KO and KO in KO, respectively). **g** Periostin (POSTN/red) immunofluorescence on callus sections after transplantation of WT PCs (GFP/green) in KO hosts. PCs express POSTN when they integrate into the callus (Merge/Yellow, left and boxes 1 and 2) and stop expressing POSTN when they differentiate (box 2, white arrowhead pointing to POSTN-negative and GFP-positive chondrocytes). Scale bar: 1 mm. **h** Histomorphometric analyses of callus, bone, and cartilage volumes at d14. Scale bar: 1 mm. SO: Safranin-O, c: cortex, ca: cartilage, cal: callus, white dashed line: bone, black dashed line: callus, merge: GFP+ cells expressing POSTN. Statistical differences between the groups were determined using Mann–Whitney test (*p < 0.05) (*n* = 3 or 4 per group). All data represent mean ± SD

To elucidate the role of the matricellular protein Periostin in the periosteal response to injury, we analyzed *Periostin* (*Postn*) expression by qRT-PCR and immunofluorescence. qRT-PCR analyses of PCs and BMSCs in un-injured tibias and tibias 3 days post-injury confirmed the specific upregulation of *Postn* gene in PCs at day 3 compared to day 0 and to BMSCs (Fig. 5a). Periostin-positive cells were detected by immunofluorescence in the cambial layer of the periosteum along the un-injured tibia (Fig. 5b). No expression of Periostin was detected in the uninjured and activated bone marrow and endosteum (Supplementary Fig. 5a). By qRT-PCR *Postn* was upregulated in sorted GFP-positive PCs isolated from *Prx1-Cre;mTmG* mice (Fig. 5b). Three days after fracture, Periostin was highly expressed in the cambial cell layer of the activated periosteum also containing CD29 expressing cells. By day 14, Periostin was expressed at the junction between late hypertrophic cartilage and bone, and by osteoblasts and osteocytes in the new bone matrix. By 28 days, Periostin was detected in the newly formed periosteum at the periphery of the ossified callus (Fig. 5b). To functionally assess the role of Periostin during bone repair, we induced tibial fractures in wild-type controls (WT) and *Periostin* KO *(*KO) mice that have been reported to exhibit post-natal growth retardation and skeletal defects including reduced trabecular bone density in long bones^48^. *Periostin* KO mice exhibit impaired bone regeneration marked by reduced callus size and bone volume throughout all stages of repair. *Periostin* KO mice failed to achieve maximum cartilage volume by day 10 followed by delayed cartilage resorption, leading to fibrosis and a non-union at day 28 (Fig. 5c, d). *Periostin* KO mice also displayed abnormal repair of unicortical bone defects that heal through direct bone formation indicating that healing via both intramembranous and endochondral ossification is affected in the absence of Periostin (Supplementary Fig. 5b, c). To assess the specific impact of *Periostin* gene invalidation on the periosteum, *GFP*-wild type or -*Periostin* KO periosteum grafts were transplanted at the fracture site of wild-type mice (Fig. 6a). *Periostin* KO periosteal grafts exhibit a decreased contribution to repair in the wild-type environment compared to WT grafts, thus Periostin is essential for periosteum activation and contribution to bone repair (Fig. 6b).

To determine if the defective periosteum response to fracture in *Periostin* KO mice was linked to impaired PCs, we isolated activated PCs from wild-type and *Periostin* KO mice 3 days after tibial fracture. The capacity of *Periostin* KO PCs to form clones was decreased compared to WT in CFE assays (Fig. 6c). *Periostin* KO PCs had impaired osteogenesis and adipogenesis compared to wild-type PCs in vitro, although chondrogenic potential was not affected (Fig. 6d). By qRT-PCR the *Periostin*-linked genes, *Lumican*, *Decorin*, *Osteoglycin*, *Thrombospondin 2,*and* Dermatopontin* upregulated in PCs in response to injury and/or encoding ECM proteins (Fig. 4i and Supplementary Table 3) showed decreased expression in *Periostin* KO PCs compared to wild type (Fig. 6e). *Endoglin 1* expression was upregulated in *Periostin* KO PCs compared to wild type and the expression of *Asporin*, *Fibrillin 1*, *Tenascin C*, *Biglycan*, *SPARC*, and *Col3a1* was not affected (Fig. 6e). The expression of these genes in *Periostin* KO BMSCs compared to wild type was not affected (Supplementary Fig. 5d). These results indicate that *Periostin* KO PCs have deficient stem cell properties in vitro and decreased expression of other ECM proteins that are normally specifically upregulated in PCs in response to injury. We then assessed the ability of wild-type PCs to rescue impaired bone healing in *Periostin* KO mice (Fig. 6f). *GFP*-wild-type trans-planted PCs showed high expression of Periostin and great capacity to integrate into cartilage (Fig. 6g). Bone volume was increased (WT in KO) compared to controls (KO in KO) and cartilage volume was decreased indicating a partial rescue of the phenotype (Fig. 6h).

### Periostin is required to maintain the pool of PCs

**Fig. 7:**
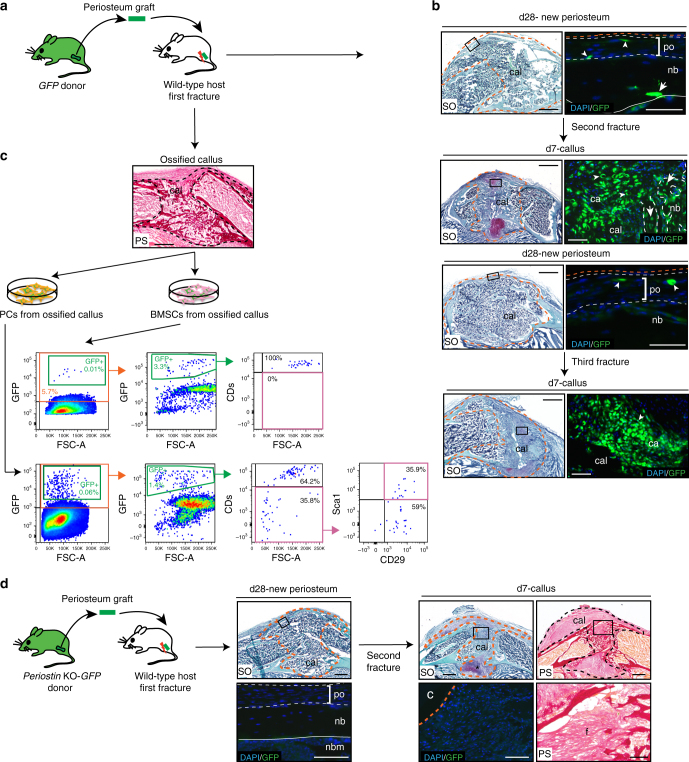
No reconstitution of the PC pool in *Postn* KO periosteum after fracture. **a** Experimental design for the isolation of periosteum graft from *GFP* donor mice and transplantation at the fracture site of wild-type hosts. **b** SO staining and DAPI/GFP immunofluorescence on longitudinal sections of mouse fractured tibias post transplantation with *GFP* periosteum graft. At d28 post fracture (d28-new periosteum), high magnification shows rare periosteum-derived GFP+ cells that integrate in the new bone to form osteocytes (white arrow) and in the new periosteum (white arrowheads). After a second fracture performed at the level of the first callus, abundant periosteum-derived GFP+ cells are found in the callus and form cartilage (white arrowheads) and bone (white arrows) (d7-callus) and few GFP+ cells reintegrate the new periosteum at d28 (white arrowheads) (d28-new periosteum). After a third fracture, periosteum-derived GFP+ cells can again form cartilage efficiently in the callus by day 7 (white arrowhead) (day 7-callus). **c** Cell sorting and FACS analyses on PCs and BMSCs isolated from ossified calluses (d14). PCs and BMSCs derived from the periosteum graft were detected based on the expression of the GFP (0.06% and 0.01%, respectively). Cell sorting was performed to enrich the population in GFP+ cells (orange box) and FACS analyses to assess the expression of hematopoietic-endothelial markers (CD11b, CD31, CD45, and CD34) and Sca1/CD29). In BMSCs cultures, GFP+ cells were all positive for hematopoietic-endothelial markers (100%). For PC cultures, we detected a population that was negative for hematopoietic-endothelial markers (35.8%) and positive for Sca1/CD29 (35.9%) (*n* = 2 or 3). **d** Transplantation of *Periostin* KO grafts into wild-type hosts. No GFP+ cells are detected in the new periosteum (d28–new periosteum), and no GFP+ chondrocytes contribute to the callus after a second injury. These *Periostin* KO grafts induced fibrosis at the fracture site of wild-type hosts (d7–callus). SO: Safranin-O/Fast Green, PS: Picro Sirius, cal: callus, po: periosteum, nb: new bone, ca: cartilage, white dashed line: periosteum (d28) or new bone (d7), orange dashed line: callus, yellow line: periosteum transplant, asterisk: cartilage formation opposite to transplant (*n* = 4). Scale bar = 1 mm

To assess more directly the role of Periostin in regulating PCs and their periosteum niche, we used periosteum transplantation (Fig. 7a). We first assessed whether periosteum contains cells that were able to repair bone and re-populate the periosteum after injury. After transplantation of GFP periosteal grafts at the fracture site of wild-type hosts (Fig. 7a)^32^, periosteum-derived GFP-positive cells largely contributed to cartilage in the callus, and rare GFP-positive cells were localized in the newly formed periosteum by d28 (Fig. 7b). To evaluate the GFP-positive cells that persisted in the callus, we isolated PCs and BMSCs from ossified calluses after transplantation. GFP-positive cells that were negative for hematopoietic and endothelial marker, and positive for Sca1/CD29, were only detected in PCs, but not in BMSCs cultures (Fig. 7c). This indicated that PCs within *GFP* donor periosteum could repopulate the newly formed periosteum. Following a second injury, these rare GFP-positive PCs could be re-activated to contribute to bone repair and were detected within cartilage and bone by day 7 (Fig. 7b). By day 28, rare GFP-positive cells were again detected in the new periosteum indicating the ability of PCs to re-populate the periosteum after the second injury (Fig. 7b). In a third cycle of injury, these re-activated PCs could again contribute to cartilage within the callus (Fig. 7b). Quantitative analyses revealed the ability of PCs to expand extensively from periosteum in the 3 cycles of injuries (Supplementary Fig. 6a). The contribution to repair of these rare PCs within the new periosteum did not decrease between the second and third injury cycles, indicating that the contribution was not due to a population of progenitor cells that would be exhausted overtime (Supplementary Fig. 6a). When we performed the same experiment with *Periostin* KO grafts into wild-type hosts, the ability of PCs to persist in the new periosteum after fracture and contribute to repair in a second injury cycle was abolished, leading to defective callus formation and fibrosis (Fig. 7d). Further, transplantation of *Periostin* KO grafts into a *Periostin* KO fracture site amplified the bone healing defect as shown by the complete absence of callus formation and bridging (Supplementary Fig. 6b as compared to the phenotype shown in Fig. 5d). Since we did not detect decreased cell proliferation in the periosteum of *Periostin* KO mice (Supplementary Fig. 6c), these results show that the *Periostin* KO phenotype is not due to a deficient proliferation, but the inability of PCs to maintain a pool of PCs in the periosteum and support bone healing in the absence of Periostin.

## Discussion

Bone regeneration is a well-orchestrated process allowing the bone to recover its proper shape and functions without the formation of scar tissue. Skeletal stem cells are activated in the early steps of bone regeneration and are the basis for this extraordinary capacity of bone to regenerate, but their endogenous origins and the mechanisms of activation are still poorly understood. Many studies have focused on the characterization of BMSCs, which are currently used in cell-based therapy approaches in orthopedics. In this study, we have identified that PCs have an enhanced capacity for cell growth and clonogenicity, as well as superior regenerative capacities compared to BMSCs. In microarray analyses, PCs have the key characteristics of SSCs, since they express “stemness” and “limb or skeletal system development” gene sets. In contrast, BMSCs are enriched in GO categories, such as “downregulation of stemness”, “bone resorption” and “immune and hematopoietic lineage”, suggesting that these cells play an indirect role during bone repair. Previous reports suggested that endogenous BMSCs are restricted to the bone marrow compartment during bone repair and indirectly stimulate healing via the secretion of growth factors^32,49^. Their role in regulating hematopoiesis and bone resorption remains to be further addressed in the context of bone repair. We show here that after transplantation at an injury site, BMSCs have reduced capacity to form cartilage and bone during skeletal regeneration compared to PCs that show great engraftment capacity further revealing the stem cell properties of PCs.

The difficulty in defining adult SSC populations and their respective functions is due to their high heterogeneity and lack of specific markers to distinguish their tissue origins. In the past few years several markers have been identified to characterize SSCs during bone development, growth, and repair^12–21^. Some of these markers may define subpopulations rather than unique populations and do not distinguish the origin of cells that are marked in the fracture callus, in particular periosteum vs. bone marrow. We show using the renal capsule transplantation approach that PCs and BMSCs are derived from a common embryonic mesenchymal lineage, but segregate in two different bone compartments during endochondral ossification to acquire different functions in adult bones. In the bone marrow compartment, BMSCs constitute the HSC niche, regulate bone turnover, and have immunomodulatory and paracrine functions during bone maintenance and repair. In the periosteum compartment, PCs are more directly involved in bone repair by forming cartilage and bone in the callus, while the role of PCs in other periosteum functions remains to be further characterized. The renal capsule model also provided strong evidence that systemic recruitment of cells during bone repair is negligible.

A hallmark of adult stem cells is their ability to self renew after injury. Self-renewal capacity has been established for other adult stem cells after tissue injury to show their ability to maintain a pool of stem cells within the same anatomical location^50^. The ability of SSCs to self renew has never been addressed in the context of bone repair until now. Sacchetti and collaborators reported that BMSCs can form de novo a bone marrow stroma organizing a hematopoietic environment surrounded by bone tissue after subcutaneous transplantation^11^. This model of heterotopic bone formation, however, does not reproduce the adequate environment to assess PC renewal after bone injury. Therefore, we designed a strategy based on periosteal grafting and subsequent injuries to specifically address the capacity of PCs to contribute to repair and repopulate the periosteum cell compartment after the repair process is completed. We show that PCs, in addition to their enhanced capacity to regenerate bone after transplantation, can maintain a pool of PCs within periosteum after injury and are mobilized again after subsequent injuries to repair bone. A similar approach based on single-muscle fiber transplantation was originally used to show extensive expansion to reform new muscle fibers in situ and self-renewal capacity of satellite cells^51^. This was not the case after transplantation of myoblasts, the muscle progenitors, which cannot contribute to repair and self-renew^52^. The fact that a small number of PCs within the initial periosteal graft can largely contribute to cartilage, give rise to rare PCs within the new periosteum and again largely contribute to cartilage in the next injury cycle indicates the presence of SSCs within the periosteum. Proliferation of progenitors could not provide a sufficient source of cells after three consecutive rounds of injury and repair, as they would disappear overtime. Furthermore, we provide evidence that this capacity of PCs to re-integrate the newly formed periosteum and contribute to repair after a second injury is abolished in the absence of Periostin without affecting the proliferation of PCs in response to injury. These data show that periosteum contains stem cells that can self renew during several injury cycles and Periostin is required for this self-renewal capacity by regulating the periosteal niche of SSCs. More investigation will be required in the future to identify specific markers for the periosteal stem cell population and follow SSC activation and self-renewal at the single-cell level within periosteum in vivo. More data will also be needed to compare the markers and tissue localization of PCs and BMSCs in mouse and human, as there are already known differences for BMSCs^11,36,53^.

An important question to elucidate is how SSCs are activated in response to injury. Our microarray data highlight several ECM proteins that are upregulated in PCs at day 3 post fracture. Within these ECM proteins, we discovered Periostin, a matricellular protein regulating cell–cell and cell–matrix interactions. Periostin is highly expressed during development and in adult tissues submitted to mechanical stress, injury or other pathological conditions^54–56^. Periostin plays a crucial role in inflammatory and tumor microenvironments^57–60^. In cancer, Periostin correlates with bad prognosis^61^ and Periostin present in the metastatic niche supports cancer stem cell self-renewal and metastatic colonization^57^. In response to bone injury, we show that Periostin and other ECM proteins linked to Periostin are upregulated in PCs and Periostin is crucial for adequate bone repair. In mice lacking *Periostin*, some of these ECM proteins are downregulated in PCs, suggesting that Periostin and Periostin-associated ECM proteins all contribute to PC activation and niche regulation in response to injury, allowing periosteal activation (Supplementary Fig. 7). Our results also re-enforce the importance of a local periosteal response at the injury site to allow callus formation and bone repair. The local activation of PCs is necessary for the bone repair process to occur and we show that a local deficiency in this PC pool in *Periostin* KO periosteum is sufficient to delay repair and induce non-union.

In conclusion, our results reveal the presence of SSCs within periosteum with higher regenerative potential compared to BMSCs. Although PCs and BMSCs derive from common mesenchymal progenitors during bone development and growth, the periosteum environment is essential to confer greater regenerative properties  to PCs. We show that PCs and their periosteum niche are two key components that act locally to allow callus formation and bone bridging for fracture consolidation. Furthermore, PCs, and the ECM components that they produce, including Periostin, are essential for periosteum activation and define the enhanced regenerative potential of PCs. Together, the roles of PCs illustrated in this study will help refocus investigation on the periosteum to elucidate numerous bone phenotypes associated with PCs rather than BMSCs defects. The skeleton possesses high regenerative capacities, yet our understanding of SSC origins, recruitment, and functions for the repair of this central organ system will necessitate more investigation of the periosteum microenvironment to find novel strategies to treat skeletal repair defects and bone diseases.

## Methods

### Mice

C57BL/6ScNj, *betaactin‐GFP (GFP)*, *Prx1-Cre*, *Rosa-tdTomato-EGFP* (*mTmG)*, *R26R*^*eYFP*^, and *R26R*^*eLacZ*^ transgenic and reporter mice were obtained from Jackson Laboratory (Bar Harbor, ME). *Prx1-Cre/ERT2-EGFP* mice were provided by Dr. S. Murakami^34^. *Periostin null* mice from Simon J. Conway laboratory were crossed with the *GFP* mice for lineage tracing^48^. The mice were bred and genotyped in our laboratory. Five to eight-week-old mice were used for in vitro experiments and more than two-month-old for in vivo experiments. All mouse primers for PCR genotyping (Supplementary Table 1) were purchased from Eurofins Scientific (Eurofins, Luxembourg). All procedures were approved by the Paris Descartes University Ethical Committee. No specific randomization methods were used for the study. However, experimental groups were homogeneous and composed of equivalent animals based on gender, age, and genotype. For each experimental group, the mice were from different litters and samples obtained from multiple experiments (>2) to generate biological replicates.

### Primary cultures of PCs and BMSCs

BMSCs and PCs were harvested from tibias and femurs of un-injured mice (d0) or from tibias 3 days post fracture (d3). The mice were killed and their hindlimbs dissected. After removing the epiphyses, bones were flushed to isolate total bone marrow cells and aBM were expanded in growth media consisting of MEMα supplemented with 20% lot-selected non-heat-inactivated FBS, 1% penicillin-streptomycin (Life Technology, Carlsbad, California), and 10 ng/ml bFGF (R&D, Minneapolis, MN). When confluence was reached, lineage depletion (CD5, CD45R (B220), CD11b, Anti-Gr-1 (Ly-6G/C), 7–4, and Ter-119 monoclonal antibodies, Miltenyi Biotec, San Diego, CA, ref. 130-090-858) was performed on aBM to obtain BMSCs that were directly used for in vitro and in vivo assays without further expansion. Although this step of lineage depletion is not standard in the literature for bone marrow cells, we chose this approach to enrich BMSCs with skeletal progenitors in order to obtain a population more comparable to PCs for the purpose of this study. Primary PCs were obtained by explant culture of the remaining flushed bones free of muscles and tendons. Explants were cultured in growth media and PCs migrated out of the explanted within 3 days. After 2 weeks, the bones were removed and PCs were trypsinized and directly used for in vitro and in vivo experiments without further expansion. To determine if our method of explant culture was optimal to retrieve PCs without contamination from the endosteum or the bone cortex, we performed the same procedure by flushing the bone marrow preceded or followed by periosteum scrapping and collagenase D digestion (0.2% collagenase D with 0.25% Trypsin/EDTA in DMEM+ 1% P/S without serum) for 1 h (Roche, Basel, CH, ref. 11088882001) prior to bone explants culture. In these conditions, the cells did not grow out of the explants (Supplementary Fig. 1a-ii and 1a-iii).

### Flow cytometry analyses and cell sorting

For flow cytometry analyses, the cells were incubated with CD31-PE-Cy^TM^7 (PECAM-1); CD45-PE-Cy^TM^7 (leukocyte common antigen, Ly-5); CD11b-PE-Cy^TM^7 (integrin αM chain); CD34-PE-Cy^TM^7 (BD Biosciences, San Jose, CA); CD29-PE, Sca1-APC (Miltenyi Biotec, San Diego, CA); and CD105-PE (eBioscience, San Diego, CA) (1:200) to label hematopoietic, endothelial, and mesenchymal lineages. For cell viability, Sytox Blue 1/1000 (Invitrogen, Thermo Fischer Scientific, Waltham, MA) was added. Analyses were performed using BD LSR Fortessa SORP (BD Biosciences, San Jose, CA). For cell sorting, the cells were resuspended in F10 media (Life Technology, Carlsbad, California) before sorting with BD FACS Aria II SORP (BD Biosciences, San Jose, CA).

### Secondary colony forming efficiency assay (CFE)

BMSCs obtained after lineage depletion of aBM and PCs obtained after explant removal were directly plated at a concentration of 400 or 2000 cells/cm^2^ in growth media consisting of MEMα supplemented with 10% FBS, 1% penicillin-streptomycin (Life Technology, Carlsbad, California), and 10ng/ml bFGF (R&D, Minneapolis, MN) for 14 days. The medium was changed every 3 days. Clones were fixed for one hour in 70% ethanol, stained with Giemsa stain (Fluka), and counted under microscope. CFE was reported in GraphPad Prism v6.0a.

### Cell-growth assay

To assess cell growth in vitro, 1.5 10^5^ BMSCs obtained after lineage depletion and PCs after explant removal were directly plated in culture dishes and cultured in growth medium. The cells were trypsinized for counting the first two days and then every two days during twelve days. Cell count was reported in GraphPad Prism v6.0a.

### In vitro osteogenic and adipogenic and chondrogenic differentiations

For each differentiation protocol, BMSCs were used following lineage depletion and PCs following explant removal without further passage. For osteogenic differentiation, the cells were plated at confluence in osteogenic medium containing MEMα with 10% FBS supplemented with 0.1 μM dexamethasone, 0.2 mM L-ascorbic acid, and 10 mM glycerol 2-phosphate disodium salt hydrate (Sigma, St. Louis, MO). The medium was changed every three days during 2–5 weeks, and the cells were stained with 0.2% alizarin red S (Sigma, St. Louis, MO). For adipogenic differentiation, the confluent cells were cultured with adipogenic medium containing MEMα with 10% FBS supplemented with 10 μg/ml insulin, 100 μM indomethacin, 0.5 mM 3-isobutyl-1-methylxanthine, and 0.1 μM dexamethasone (Sigma, St. Louis, MO). The medium was changed every 3 days during 3 weeks and the cells were stained with Oil Red O solution (Sigma, St. Louis, MO). Nuclei were counterstained with Harris hematoxylin (DiaPath, Martinengo, Italy). Pictures of lipid droplets were taken under light microscopy using Leica DM IRB light microscope and LAS v4.3 software (Leica Microsystems Inc, Buffalo Grove, IL). For chondrogenic differentiation, the cells were resuspended at a concentration of 5.10^5^ cells in 200 μl of growth media and plated as micromass. After 2 h at 37 °C, the cells were covered with chondrogenic medium containing DMEM with 10% FBS supplemented with 0.1 μM dexamethasone, 100 μg/ml sodium pyruvate, 40 μg/ml L-proline, 50 μg/ml L-ascorbic acid, 50 mg/ml ITS, and 10 ng/ml TGFβ1. The medium was changed every 3 days during 1–2 weeks and the cells were stained with Alcian blue (Sigma, St. Louis, MO).

### Wound healing assay

Wound healing assay was performed to assess migration capacity of cells in vitro. Forty-eight hours before the assay, BMSCs obtained after lineage depletion and PCs after explant removal were directly plated in culture inserts in μ-slide 8 well ibiTreat (Biovalley) and cultured in growth media. Before starting the assay, culture inserts were removed allowing a clear separation between two migration fronts (wound). A volume of 10 μM of Cytosine β-D-arabinofuranoside hydrochloride (Sigma, St. Louis, MO) was added in the medium to inhibit cell mitosis. Wound healing was recorded every 10 minutes over 50 to 72 h using videomicroscopy (Nikon Eclipse Ti-E). Data were analyzed with ICY software (bioimageanalysis.org) and reported in GraphPad Prism v6.0a.

### Fractures and cell transplantations

Closed non-stabilized and open non-stabilized tibial fractures were performed in the mid-diaphysis under anesthesia and analgesia^2,32^. For all surgeries, mice were anesthetized with an intraperitoneal injection of Médétomidine (1 mg/kg) and Kétamine (50 mg/ml) and received a subcutaneous injection of Buprenorphine (0,1 mg/kg) for analgesia. For closed fractures, the tibia was placed on the fracture jig and 460 g weight was dropped from 14 cm to create a closed, transverse fracture by three-point bending, which was confirmed by radiography. Opened non-stabilized tibial fractures were produced by osteotomy. The anterior tibial surface was exposed by separating the bone from the surrounding muscles. Three holes were drilled in the tibial cortex using a 0.4 mm drill bit and the bone was cut to create the fracture. After surgery, the mice were revived with a subcutaneous injection of Atipamezole (1 mg/kg) and were allowed to move freely. The mice then received a second dose of analgesic 12–24 h after surgery and subsequent doses as needed. For cell transplantations at the fracture site, 100,000 cells were embedded in a fibrin gel using a Tissucol^®^ kit (Baxter, France TISSEEL, composed of human fibrinogen 15 mg/ml and thrombin 9 mg/ml) and the cell pellet was transplanted at the time of fracture^62^.

### Cortical bone defects

To assess the impact of *Periostin* deficiency on bone healing through intramembranous ossification, we performed unicortical bone defects (without breaking the bone) on wild-type and *Periostin* KO mice as previously described^24^. Briefly, after anesthesia and analgesia, the tibial surface was exposed, and a hole (1 mm in diameter) was drilled into one cortex without drilling into the opposite cortex. After surgery, the mice were revived as indicated above.

### Periosteum grafting

Periosteum grafts isolated from the tibia *GFP* or *Prx1-Cre*;*mTmG* donor mice were transplanted at the site of open non-stabilized tibial fractures in 10-week-old wild-type or *Periostin* KO host mice^32^. For assessment of long-term engraftment, successive fractures at one-month interval were performed at the site of initial bone graft in 5-week-old hosts and fracture calluses were collected at days 7 and 28. For BMSCs and PCs cultures, day 14 ossified calluses were retrieved by dissection. BMSCs were obtained by flushing the bone marrow cells from the fracture calluses followed by adherence and lineage depletion calluses as described above. PCs were cultured by explant cultures of remaining ossified parts of the fracture calluses. Fracture callus pieces were carefully cleaned and placed in culture periosteum facing down. PCs and BMSCs were used for cell sorting and FACS analyses.

### Renal capsule transplantation

Femoral grafts containing cartilage anlage surrounded by perichondrium were isolated from E14.5 donor embryos, transplanted in the renal capsule of adult host mice, and allowed to develop for 8 weeks to form fully mature bones^43^. PCs and BMSCs were isolated as described above from bones derived from *Prx1-Cre*;*YFP*^*fl/+*^ donors transplanted in C57BL/6ScNj (wild type) hosts and analyzed via flow cytometry as indicated above. For lineage analyses during bone repair, the bones derived from *Prx1-Cre*;*LacZ*^*fl/+*^donors transplanted in wild-type hosts were fractured by osteotomy under anesthesia by exposing the kidney capsule and collected 7 or 14 days post fracture for lineage tracing using Xgal/TRAP staining on tissue sections as previously described^63^. In control samples, the genotypes of the donor (wild type) and host (*Prx1-Cre*;*YFP*^*fl/+*^or *Prx1-Cre*;*LacZ*^*fl/+*^) were reversed.

### Histomorphometry and cell-lineage analyses

The mice were killed at specified time points post fracture. Tibias were fixed in 4% paraformaldehyde, decalcified in 19% EDTA, and processed for histomorphometric analyses of callus, cartilage, and bone on Safranin-O (SO) and Trichrome (TC) stained sections^62,64^. Picrosirius staining was performed on adjacent sections to visualize bone and fibrous tissue. For quantitative analyses of GFP-transplanted cells in fracture calluses, GFP signal was analyzed on sections adjacent to Safranin-O and Trichrome using a Zeiss Imager D1 AX10 light microscope and ZEN software (Carl Zeiss Microscopy GmbH, Gottinger, Germany).

### Immunofluorescence and immunohistochemistry

For PCNA immunofluorescence, the sections were rehydrated, post fixed in 4% paraformaldehyde for 10 min, treated with methanol for 10 min, permeabilized with 0.25% TritonX-100 in PBS, and blocked with 5% Goat serum in 0.25% tritonX-100 in PBS for 15 min. The sections were then incubated with primary antibody rabbit anti mouse PCNA 1:800 (Cell Signaling, Danvers, MA ref. 13110 s) or non-immune rabbit IgG as negative control (Invitrogen, Thermo Fischer Scientific, Waltham, MA ref. 10500 C) O/N at 4 °C. The sections were washed and incubated with secondary antibody Alexa 546 goat anti rabbit 1:500 (Invitrogen, Thermo Fischer Scientific, Waltham, MA ref.11010) in 5% goat serum for one hour at RT, and mounted with Fluoromount-G™ with DAPI (eBioscience, San Diego, CA).

For Cleaved Caspase 3 immunofluorescence, the sections were rehydrated, post fixed in 4% paraformaldehyde for 10 min, permeabilized with 0.25% TritonX-100 in PBS, and blocked with 5% Goat serum in 0.25% tritonX-100 in PBS for one hour. The sections were then incubated with primary antibody rabbit anti mouse Cleaved Caspase 3 1:200 (Cell Signaling, Danvers, MA ref. 9661) or non-immune rabbit IgG as negative control (Invitrogen, Thermo Fischer Scientific, Waltham, MA ref. 10500 C) O/N at 4 °C. The sections were washed and incubated with secondary antibody Alexa 546 goat anti rabbit 1:800 (Invitrogen, Thermo Fischer Scientific, Waltham, MA ref.11010) in 5% goat serum for one hour at RT and mounted with Fluoromount-G™ with DAPI (eBioscience, San Diego, CA).

For Periostin immunofluorescence, the sections were rehydrated, blocked with 5% donkey serum in PBS one hour at room temperature (RT), and incubated with primary antibody goat anti mouse Periostin 1:400 (R&D, Minneapolis, MN ref. AF2955) or non-immune goat IgG as negative control (Life Technology, Carlsbad, California ref. 026202) overnight (O/N) at 4 °C. The sections were washed and incubated with secondary antibody Alexa 488 donkey anti goat or Alexa 546 donkey anti goat 1:500 (Invitrogen, Thermo Fischer Scientific, Waltham, MA ref. A11055 or ref. A11056) for one hour at RT. Slides were mounted with Fluoromount-G™ with DAPI (eBioscience, San Diego, CA).

For CD29 immunofluorescence, the sections were rehydrated, post fixed in 4% paraformaldehyde for 10 min, permeabilized with 0.25% TritonX-100 in PBS, and blocked with 5% donkey serum in PBS for 15 min. The sections were incubated with primary antibody goat anti mouse integrinβ1 5 μg/ml (R&D, Minneapolis, MN, ref. AF2405) or non-immune goat IgG as negative control (Life Technology, Carlsbad, California ref. 026202) O/N at 4 °C. The sections were washed and incubated with secondary antibody Alexa 546 donkey anti goat or Alexa 647 donkey anti goat 1:500 (Invitrogen, Thermo Fischer Scientific, Waltham, MA) in 5% donkey serum for one hour at RT and mounted with Fluoromount-G™ with DAPI (eBioscience, San Diego, CA).

For BrdU immunochemistry, the mice were beforehand injected with 50 mg/kg of BrdU (Sigma, St. Louis, MO ref. B5002) in 5% DMSO and their hindlimbs were harvested three hours later and processed as previously described. The sections were dehydrated in ethanol baths and antigen retrieval was performed using 2 N HCl in 0.5% Triton-X100 for 30 min at RT. Endogenous peroxidase activity was blocked using 3% H_2_O_2_ in PBS for 10 min. Sections were blocked in 5% goat serum in PBS for 1 h and incubated with primary antibody rat anti mouse BrdU 1:200 (Abcam, Cambridge, UK ref. Ab6326) or no primary antibody as negative control, O/N at 4 °C. The sections were washed and incubated with secondary antibody biotin goat anti rat 1:500 (Jackson ImmunoResearch, West Grove, PA ref. 112066072) for 1 h at RT. After washing in PBS, the sections were incubated in Streptavidin-HRP 1:100 (BD Biosciences, San Jose, CA ref. 554066) for 30 min at RT. Finally, signal was revealed using DAKO kit (Agilent, Santa Clara, CA ref. K3467) and counterstained with 5% Methyl green. BrdU+ cells were counted under microscope and reported in GraphPad Prism v6.0a.

### RNA isolation and qRT-PCR

Total mRNA extraction from cells was performed using RNeasy Plus Mini Kit (Qiagen, Germantown, MD) and following manufacturer’s instructions. The concentration of extracted RNA was confirmed using a NanoDrop 2000 UV-Vis Spectrophotometer (Thermo Scientific, Wilmington, DE). All mouse primers (Supplementary Table 2) were purchased from Eurofins Scientific (Eurofins, Luxembourg). cDNA synthesis was performed using Superscript III RT, RNaseOUT, Ribonuclease inhibitor, Oligo(dT)12–18, 10 mM dNTP mix, 5X first-strand buffer, and 0.1 M DTT, following manufacturer’s instructions (Thermo Fischer Scientific, Waltham, MA). Real-time PCR was performed using SYBR™ Green PCR Master Mix and detected using 7300 Real-Time PCR System (Thermo Fischer Scientific, Waltham, MA). Mouse *GAPDH* was used as an internal control for all genes.

### Microarray analyses

BMSCs and PCs were isolated and purified from uninjured tibia (d0) (*n* = 4) and tibia 3 days post fracture (*n* = 4). The cells were harvested and total RNA was extracted using Rneasy Plus mini Kit (Qiagen). RNA quality was assessed using Agilent Model 2100 Bioanalyzer (Agilent Technologies). Gene expression analyses were performed using GeneChip Mouse 430 2.0 Array (Affymetrix). Fluorescence data were imported into Affymetrix Expression Console and R Bioconductor analysis software. Data were normalized with RMA method, groups were compared by Student’s *t-*test and the results were filtered at *p-*value ≤5% and fold change ≥1.2. Hierarchical clustering was performed using MultiExperiment Viewer software (MeV)^65,66^. Gene Set Enrichment Analysis (GSEA) analysis was performed using all normalized probes on “curated gene set” and “GO gene set” collections of the Molecular Signatures Database v5.2 according to^67,68^. *Postn*-linked gene list was built using STRING database^69^ (http://string-db.org/cgi/input.pl?UserId=5bskVvnWAJdi&sessionId=hMz9XrOQGQ4P&input_page_show_search=on). We used the following parameters: active interaction sources: all checked; minimum required interaction score: 0.4; maximum number of interactors to show: 1^st^ shell: 100; and 2^nd^ shell: 20. We obtained a list of 93 genes (Supplementary Table 3).

### Statistical analyses

Statistical significance was determined with two-sided Mann–Whitney test and reported in GraphPad Prism v6.0a. *P-*values were determined as follows: *^,$^*p* ≤ 0.05; **^,$$^*p* < 0.001; ***,^$$$^*p* < 0.0005. All samples were included except for fractures that were proximal and/or distal or comminuted fractures. All analyses were performed using a blind numbering system.

### Data availability

The microarray data have been deposited in the ArrayExpress database under the accession number E-MTAB-6417. All other data are available in the article and in the supplementary information files.

## Electronic supplementary material

Supplementary Information
