# Supplementary material for: Periosteum contains skeletal stem cells with high bone regenerative potential controlled by Periostin
Source: Nat Commun. 2018 Feb 22;9:773. doi: 10.1038/s41467-018-03124-z (PMC5823889; doi:10.1038/s41467-018-03124-z)
Supplement: Supplementary file 1 — Supplementary Information [file 41467_2018_3124_MOESM1_ESM.pdf]

Supplementary Information

Periosteum contains skeletal stem cells with high bone regenerative potential controlled by Periostin

Duchamp de Lageneste et al.

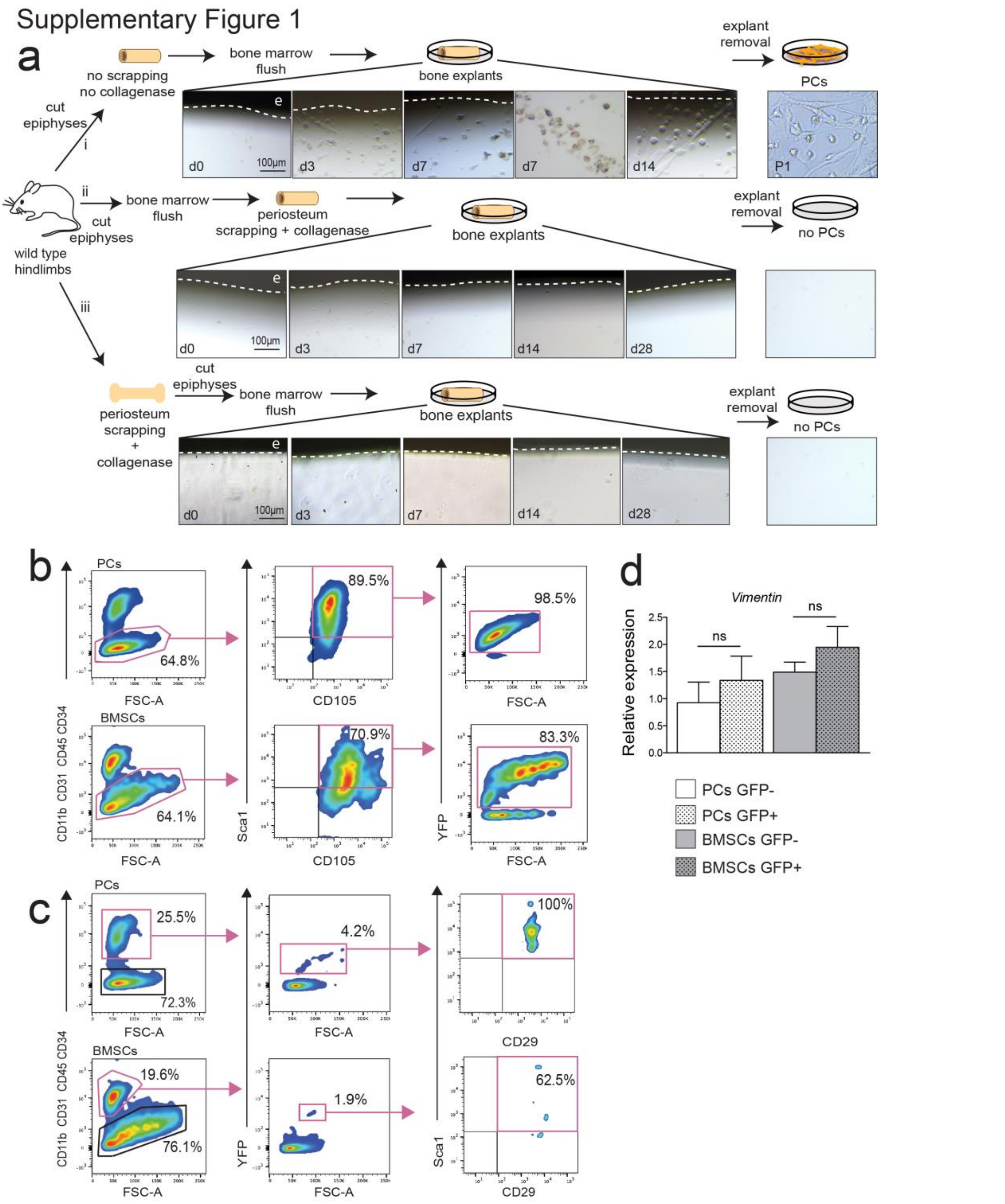

Supplementary Figure 1. Optimization of periosteal cell (PC) cultures and flow cytometry analyses of Prx1-derived PCs and BMSCs. (a) Experimental design for periosteal cell (PC) cultures. After epiphyses removal, bone marrow was flushed and all muscles and tendons were removed. Bone explants were directly placed in culture dishes to allow PCs to migrate out of the periosteum (a-i, protocol used for all experiments). As controls, to show that PCs were derived only from periosteum and not from bone marrow compartment and/or bone itself, periosteum was scrapped followed by digestion of the explant with collagenase D (a-ii shows that no cells come from the bone cortex, a-iii shows that no cells come from bone cortex and bone marrow). No PCs can grow in these conditions. (b-c) Flow cytometry analyses of PCs and BMSCs isolated from *Prx1-Cre;YFP*$^{fl/+}$ mice. (b) PCs and BMSCs negative for endothelial/hematopoietic markers (CD31, CD11b, CD34, CD45) and double-positive for Sca1/CD105 are mostly YFP+ (derived from Prx1 lineage). (c) The population positive for endothelial/hematopoietic markers (25.5% in PCs and 19.6% in BMSCs) are largely negative for YFP (not derived from Prx1- lineage). (d) Quantitative RT-PCR analysis of *Vimentin* gene expression on FACS sorted GFP-positive and GFP-negative PCs and BMSCs isolated from *Prx1-Cre;mTmG* mice. Statistical differences between the groups were determined using Mann-Whitney test (*$p \leq 0.05$, **$p<0.001$, ***$p<0.0005$) (n=3). All data represent mean ± SD.

Supplementary Figure 2

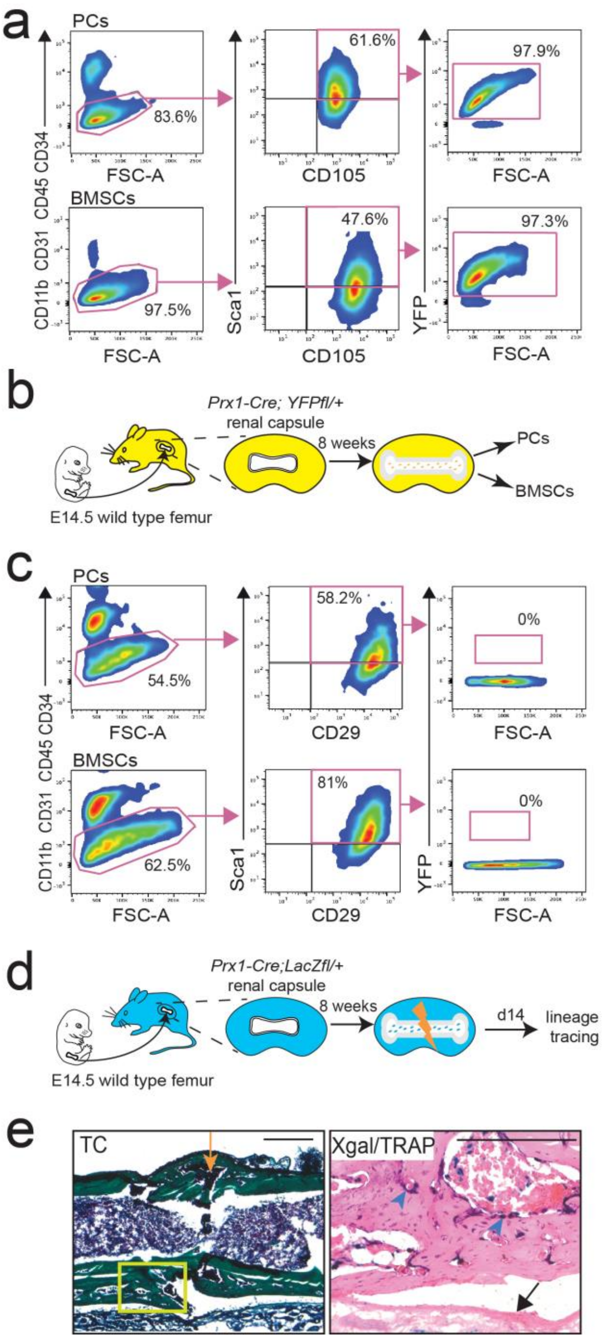

Supplementary Figure 2. Lineage tracing of Prx1-lineage during skeletal development and repair in the renal capsule environment - Absence of the host contribution. (a) Flow cytometry analyses of PCs and BMSCs isolated from *Prx1-Cre;YFP*$^{fl/+}$ mature skeletal elements grown under renal capsule of wild type hosts as shown in Figure 2a. PCs and BMSCs that are negative for endothelial/hematopoietic markers (CD31, CD11b, CD34, CD45) and positive for Sca1/CD105 are YFP-positive donor-derived. (b) Experimental design for renal capsule transplantations of femoral cartilages isolated from E14.5 wild type embryos and transplanted under the renal capsule of *Prx1-Cre;YFP*$^{fl/+}$ adult hosts. PCs and BMSCs were isolated from mature skeletal elements 8 weeks post-transplantation as described in Fig. 1a. (c) Flow cytometry analyses show that PCs and BMSCs that are negative for endothelial/hematopoietic markers (CD31, CD11b, CD34, CD45) and positive for Sca1/CD29 are YFP-negative donor-derived. No contribution of the host-derived Prx1 lineage can be detected. (d) Experimental design for cell-lineage analyses during bone regeneration in renal capsule. Femoral cartilages were isolated from E14.5 wild type embryos and transplanted under the renal capsule of *Prx1-Cre*$^{+/-}$*;LacZ*$^{fl/+}$ hosts. After 8 weeks, mature femurs underwent osteotomy and were collected at d14 post-fracture for cell lineage tracing. (e) TC and Xgal/TRAP double staining on longitudinal sections of wild type fractured femurs in *Prx1-Cre*$^{+/-}$*;LacZ*$^{fl/+}$ hosts showing no LacZ+ osteocytes in the callus confirming no systemic contribution of the host Prx1 lineage to repair (blue arrowheads, LacZ+/TRAP+ bone lining osteoclasts with endogenous beta-galactosidase activity). TC: Masson's trichrome, TRAP: Tartrate resistant acid phosphatase, orange arrow: fracture site, black arrow in (e): periosteum, blue arrowhead: osteoclasts.

Supplementary Figure 3

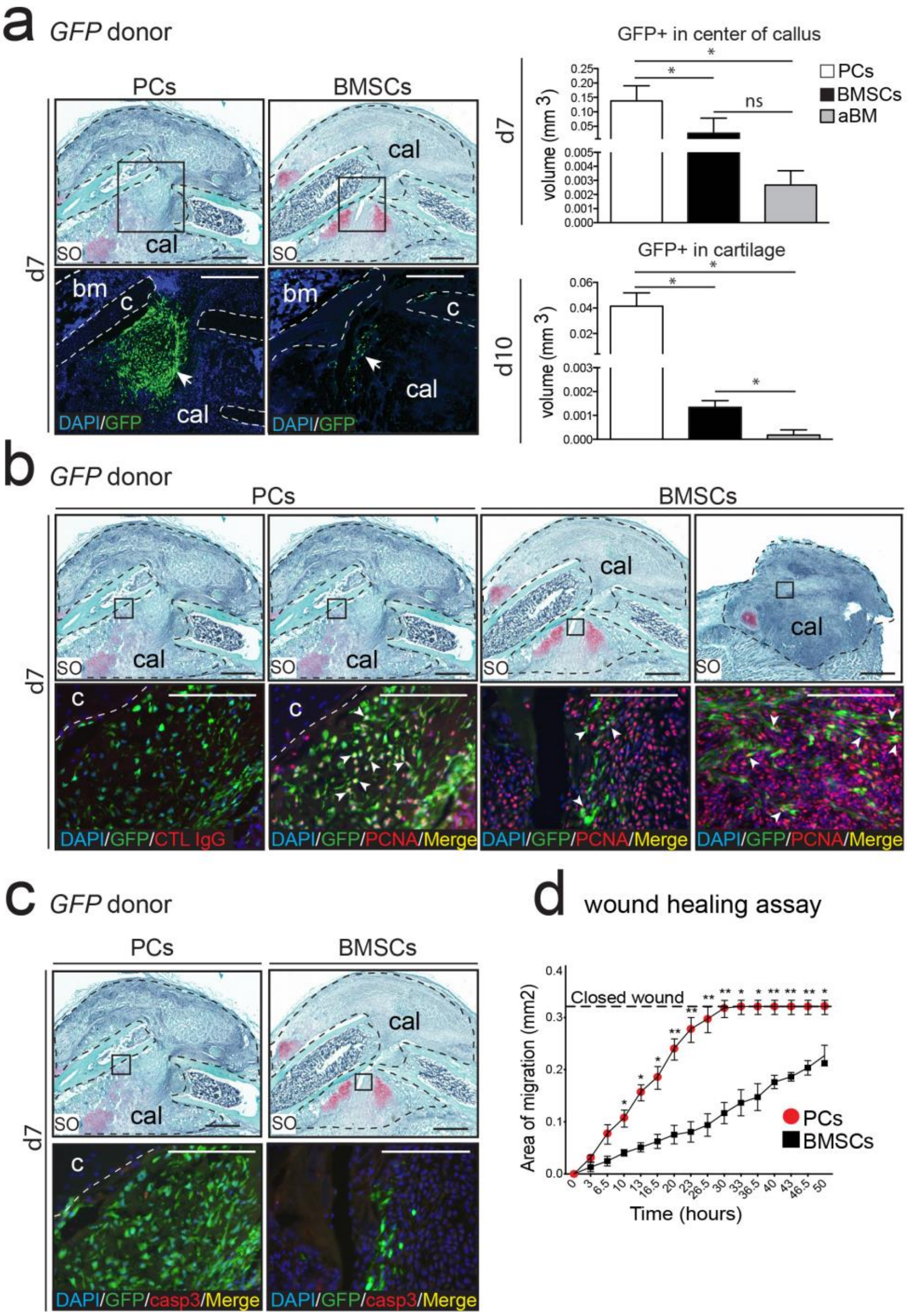

Supplementary Figure 3. Proliferation, cell death and cellular contribution of PCs and BMSCs to bone repair in vivo and migration potential in vitro. (a) Lineage tracing of PCs (left column) and BMSCs (right column) derived from GFP donors in the fracture callus after transplantation in wild type hosts. SO staining and DAPI/GFP immunofluorescence on longitudinal sections at day 7 (d7) shows more PCs than BMSCs in the center of the fracture callus (GFP+ pointed by white arrows) (n=5 per group). Histomorphometric analyses show similar contribution of BMSC and aBM to bone callus formation at day 7 and increased contribution of BMSCs to cartilage at day 10 compared to aBM. (b) PCs and BMSCs proliferate in the callus after transplantation. Immunofluorescence anti-PCNA shows PCNA/GFP double positive PCs in the center of the callus. BMSCs in the center of the callus are also PCNA/GFP double positive (BMSCs left panel) but most of transplanted/proliferating BMSCs remain at the periphery of the callus (BMSCs right panel). (c) Immunofluorescence anti-Cleaved Caspase 3 (casp3) shows no cell death at day 7 after transplantation of either PCs or BMSCs. (d) Migration potential of PCs and BMSCs. In vitro wound healing assay shows PCs closing the wound 30 hours after plating while BMSCs were still migrating (n=3 per group). Black dashed line: callus, White dashed line: bone cortex, white arrowheads point to PCNA+ cells, Merge: GFP/PCNA double positive cells (b) or GFP/cleavedCaspase3 double positive cells (c), SO: Safranin-O/Fast Green, cal: callus, c: cortex, bm: bone marrow. Scale bar: 1mm. Statistical differences between the groups were determined using Mann-Whitney test (*$p \leq 0.05$, **$p < 0.001$, ***$p < 0.0005$) (n= 3 to 5). All data represent mean ± SD.

Supplementary Figure 4

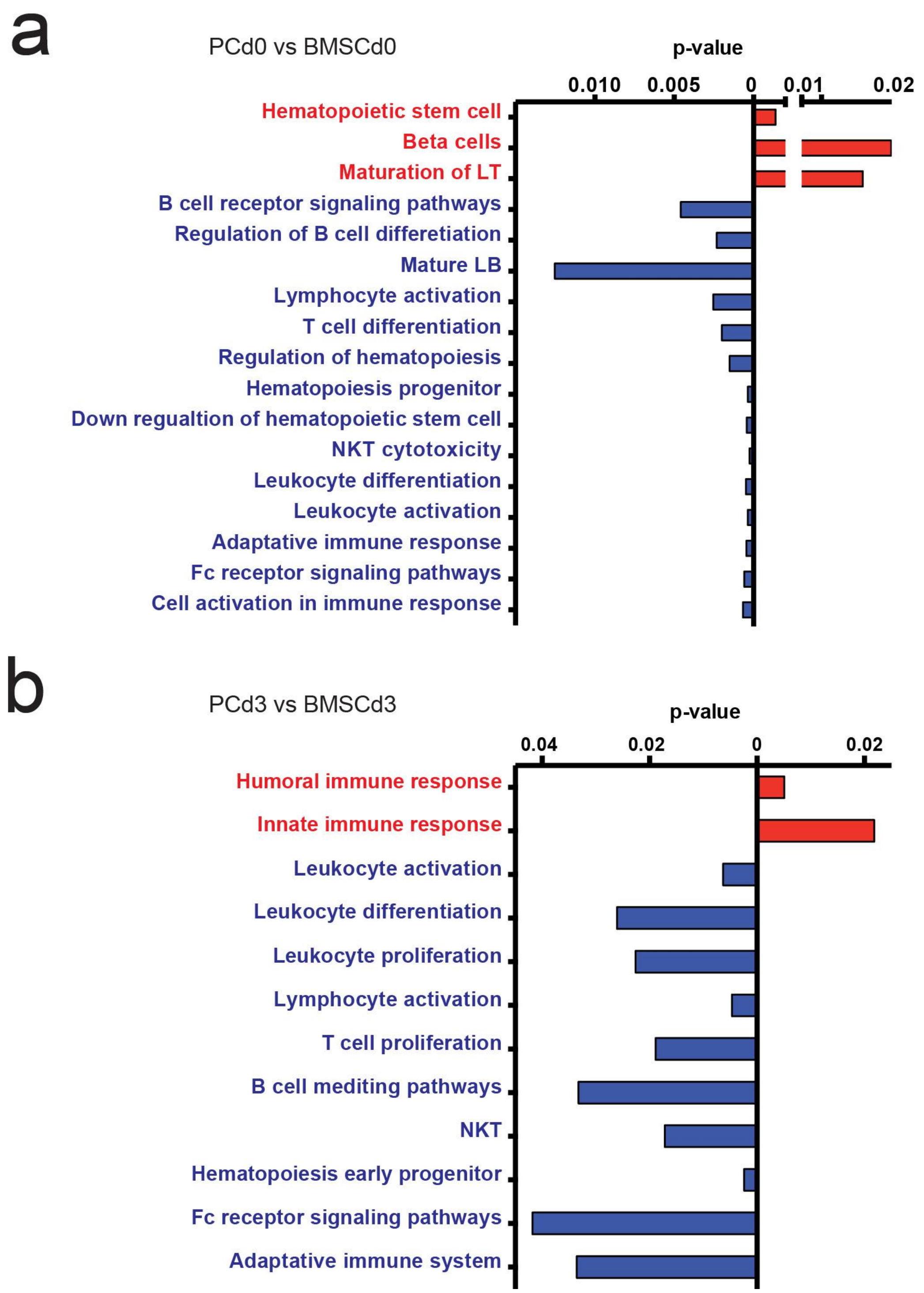

Supplementary Figure 4. BMSCs are highly enriched in hematopoietic and immune functions compared to PCs at day0 and day3. (a) GSEA analysis comparing PCd0 versus BMSCd0. (b) GSEA analysis comparing PCd3 versus BMSCd3. Gene sets enriched in PCs are shown in red and those enriched in BMSCs are shown in blue.

Supplementary Figure 5

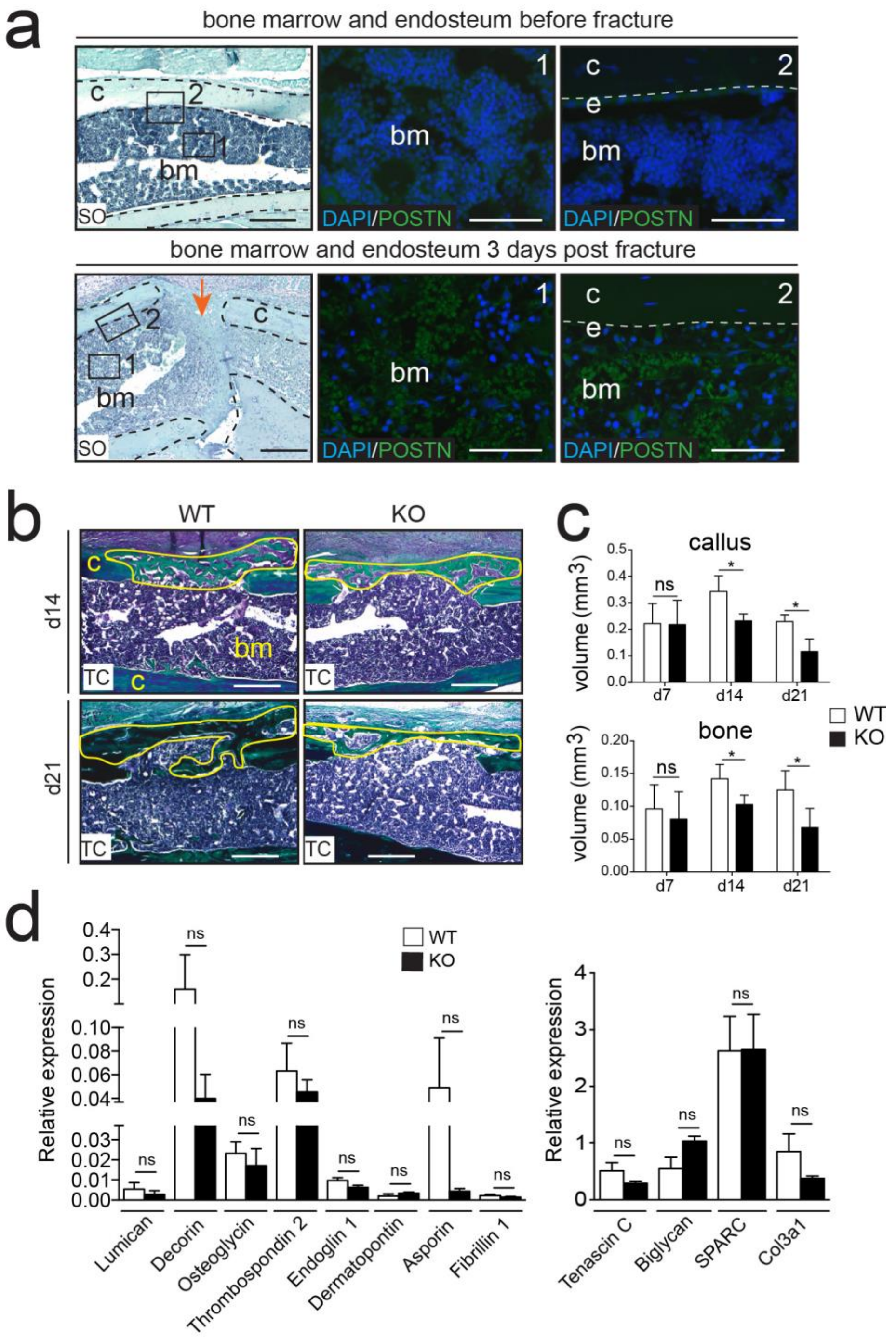

Supplementary Figure 5. Expression analyses of *Periostin* and *Periostin*-linked genes in bone marrow during healing via endochondral ossification and impaired bone healing via intramembranous ossification in *Postn* KO mice. (a) SO staining and DAPI/POSTN immunofluorescence on wild type longitudinal tibia sections showing the absence of Periostin (POSTN) expression in the bone marrow and endosteum of un-injured and injured tibias at day 3 post non-stabilized fractures as compared to high expression in periosteum in Figure 5a (immunofluorescences correspond to box areas in SO). Scale bar: 0.5mm. (b) TC staining on longitudinal sections of mouse tibia at d14 and d21 post cortical defect in wild type (WT) and *Periostin* KO (KO) mice (n=4). Scale bar: 0.5 mm. (c) Histomorphometric analyses show impaired callus and bone formation at d14 and d21 post cortical defect in *Periostin* KO mice compared to wild type littermates. (d) Quantitative RT-PCR analyses of *Periostin* linked genes *Lumican, Decorin, Osteoglycin, Thombospondin 2, Endoglin 1, Dermatopontin, Asporin, Fibrillin 1, Tenascin C, Biglycan, SPARC, Col3a1* in WT- and KO-BMSCs shows no downregulation in KO-BMSCs compared to WT at day 3 post non-stabilized fracture (as compared to downregulation in PCs in Figure 6e) (n=3). SO: Safranin-O/Fast Green, TC: Masson's trichrome, bm: bone marrow, c: cortex, e: endosteum. Black dashed line and white dashed line: cortex, yellow line: new bone formation. Statistical differences between the groups were determined using Mann-Whitney test (*p≤0.05). All data represent mean ± SD.

Supplementary Figure 6

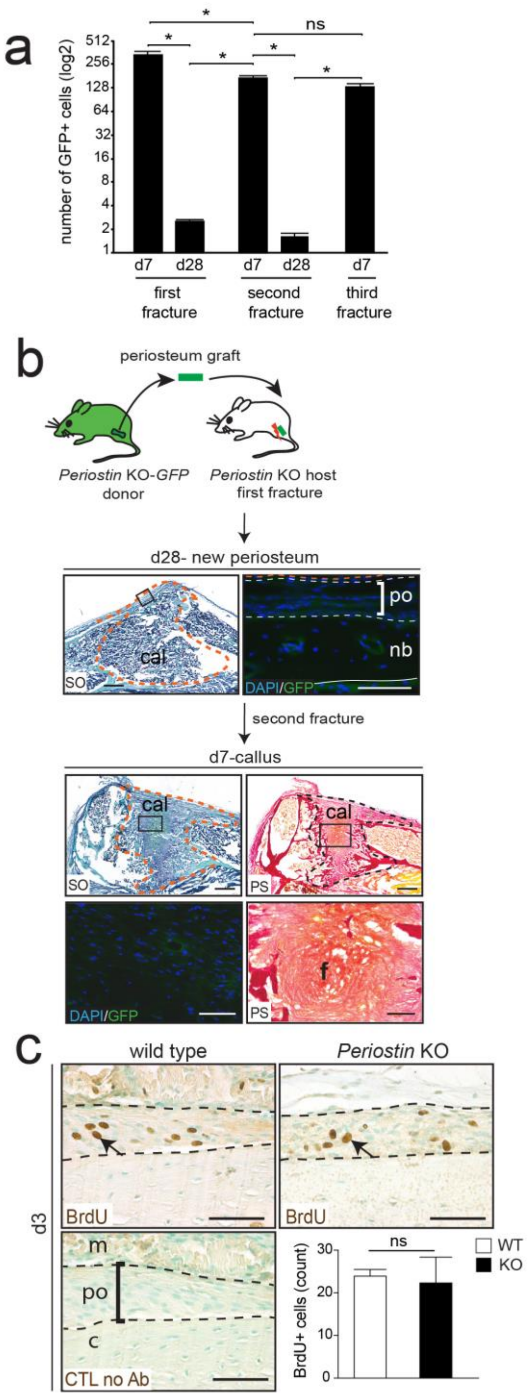

Supplementary Figure 6, related to Figure 7. The ability of periosteal cells to form cartilage and to colonize the new periosteal niche after periosteum transplantation is impaired in the absence of Periostin. (a) Quantitative analyses of GFP periosteal grafts from Figure 7a-c show the ability of periosteal cells derived from the GFP periosteal graft to expand extensively from periosteum in 3 rounds of injuries. The number of GFP+ cells is high in the cartilage in response to the fracture (d7) compared to the rare GFP+ cells found in the new periosteum at d28. (b) Transplantation of *Periostin* KO GFP graft into *Periostin* KO hosts shows that the absence of Periostin abolishes the ability of periosteal cells to re-populate the periosteal niche (d28-new periosteum, no GFP+ cells), and to form cartilage after a second injury (d7-callus, no GFP+ cells). As a consequence, the pseudarthrosis phenotype is observed as early as day 7 *Periostin* KO hosts causing a more severe bone repair defect compared to the first injury (d7, fibrosis showed by Picrosirius Staining, PS). (c) Immunohistochemistry anti-BrdU shows that cell proliferation is not affected in the periosteum in the absence of Periostin (black arrows: BrdU+ cells). SO: Safranin-O/Fast Green, cal: callus, po: periosteum, nb: new bone, m: muscle, white dashed line: periosteum, orange and black dashed lines: callus, black dashed line in (c): periosteum, n=2 to 4 per group. Scale bar= 1mm. Statistical differences between the groups were determined using Mann-Whitney test (*$p \leq 0.05$) (n=3). All data represent mean ± SD.

Supplementary Figure 7

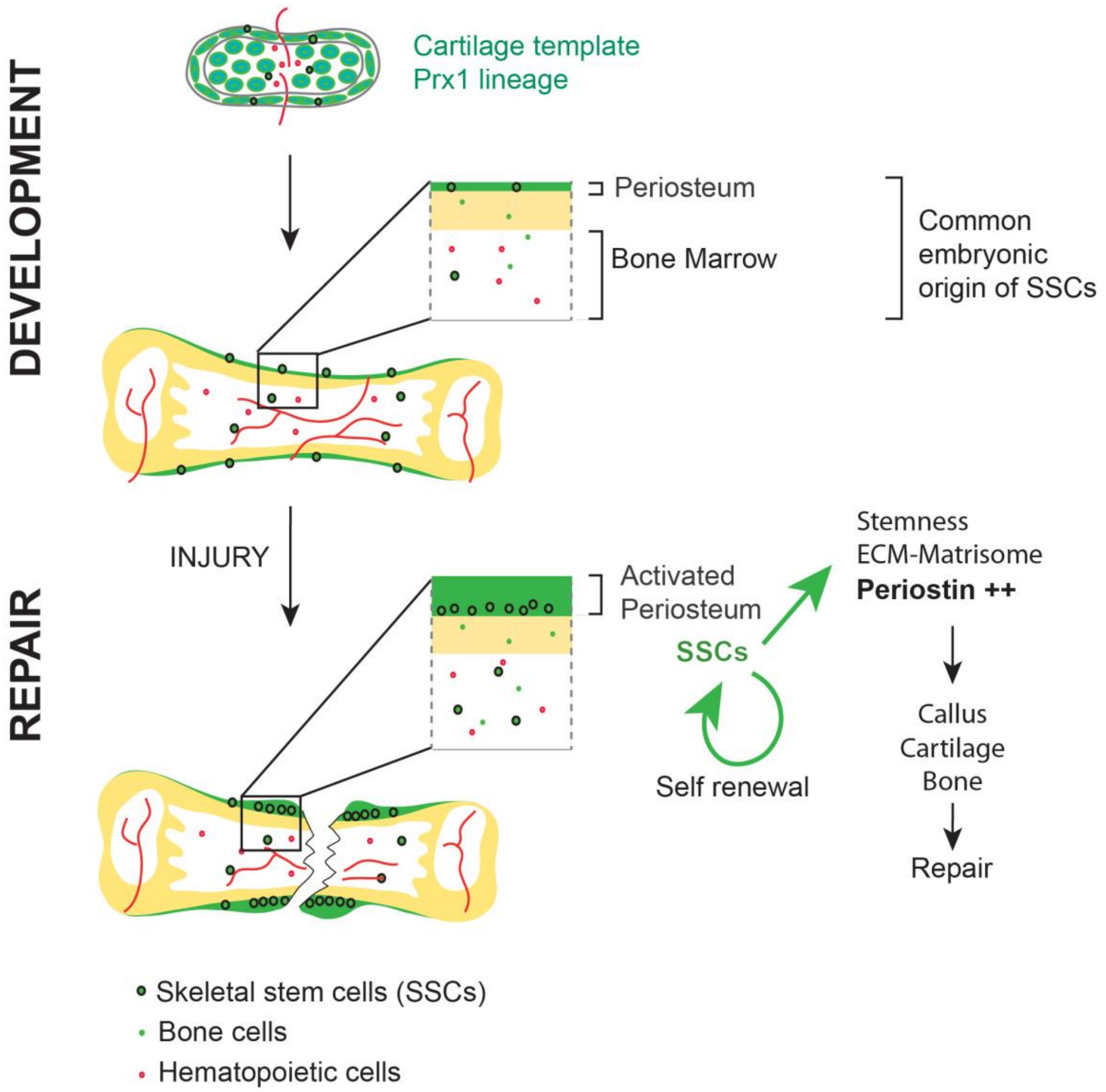

Supplementary Figure 7. Model of skeletal stem cell (SSC) origins and functions during bone regeneration. SSCs in periosteum and bone marrow share a common embryonic origin and derive from the Prx1-mesenchymal lineage. SSCs in periosteum have increased regenerative potential compared to bone marrow and self renew within periosteum after bone injury. Extracellular matrix proteins including the matricellular protein Periostin mark the periosteum response to injury and Periostin overexpression in periosteum after bone injury is required for periosteum activation and bone repair.

Supplementary Table 1. Primers for PCR genotyping

name	Primers
mouse *Prx1-Cre*	5’-CCTGGAAAATGCTTCTGTCCG-3’
	5’-CAGGGTGTTATAAGCAATCCC-3’
mouse *mTmG*	5’-CTCTGCTGCCTCCTGGCTTCT-3’
	5’-CGAGGCGGATCACAAGCAATA-3’
	5’-TCAATGGGCGGGGGTCGTT-3’
mouse $R26R^{eYFP}$	5’-AAGACCGCGAAGAGTTTGTC-3’
	5’-GGAGCGGGAGAAATGGATATG-3’
	5’-AAAGTCGCTCTGAGTTGTTAT-3’
mouse $R26R^{eLacZ}$	5'-AAAGTCGCTCTGAGTTGTTATCA-3'
	5'- GTGGGAAGTCTTGTCCCTCC -3'
	5'-CTTCCATTTGTCACGTCCTGC-3'
mouse *Periostin*	5’-AGTGTGCAGATGTTTGCTTG-3’
	5’-ACGAAATACAGTTTGGTAATCC-3’
	5’-CAGCGCATCGCCTTCTATCG-3’

Supplementary Table 2. Primers for qPCR.

Gene name	Primer pair	Primer sequence
mouse *PDGFRα*	*PDGFRα* for	5’-AGAGTTACACGTTTGAGCTGTC-3’
	PDGFRα rev	5’-GTCCCTCCACGGTACTCCT-3’
mouse *Gremlin 1*	*Gremlin 1* for	5’-AAGCGAGATTGGTGCAAAACT-3’
	Gremlin 1 rev	5’-GAAGCGGTTGATGATAGTGCG-3’
mouse *Cxcl12*	*Cxcl12* for	5’-GAGCCAACGTCAAGCATCTG-3’
	Cxcl12 rev	5’-CGGGTCAATGCACACTTGTC-3’
mouse *Leptin Receptor*	*Leptin R* for	5’-ATGTGCCCTTCCGATATACAACC-3’
	Leptin R rev	5’-CGTGTCATCCACTAATCTTCTGG-3’
mouse *Nestin*	*Nestin* for	5’-TCCCTTAGTCTGGAAGTGGCTA-3’
	Nestin rev	5’-GGTGTCTGCAAGCGAGAGTT-3’
mouse *NG2*	*NG2* for	5’-GGGCTGTGCTGTCTGTTGA-3’
	NG2 rev	5’-TGATTCCCTTCAGGTAAGGCA-3’
mouse *Vimentin*	*Vimentin* for	5’-CTGCTTCAAGACTCGGTGGAC-3’
	Vimentin rev	5’-ATCTCCTCCTCGTACAGGTCG-3’
mouse *Periostin*	*Postn* for	5’-CCTGCCCTTATATGCTCTGCT-3’
	Postn rev	5’-AAACATGGTCAATAGGCATCACT-3’
mouse *Lumican*	*Lumican* for	5’-CTCTTGCCTTGGCATTAGTCG-3’
	Lumican rev	5’-GGGGGCAGTTACATTCTGGTG-3’
mouse *Decorin*	*Decorin* for	5’-TCTTGGGCTGGACCATTTGAA-3’
	Decorin rev	5’-CATCGGTAGGGGCACATAGA-3’
mouse *Osteoglycin*	*Osteoglycin* for	5’-ACCATAACGACCTGGAATCTGT-3’
	Osteoglycin rev	5’-AACGAGTGTCATTAGCCTTGC-3’
mouse *Thrombospondin 2*	*Thbs2* for	5’-CTGGGCATAGGGCCAAGAG-3’
	Thbs2 rev	5’-GCTTGACAATCCTGTTGAGATCA-3’
mouse *Asporin*	*Asporin* for	5’-AAGGAGTATGTGATGCTACTGCT-3’
	Asporin rev	5’-ACATTGGCACCCAAATGGACA-3’
mouse *Fibrillin 1*	*Fibrillin 1* for	5’-GGACGCCAATTTGGAGGCT-3’
	Fibrillin 1 rev	5’-CTTTCAGCGCATCGTGTCCT-3’
mouse *Tenascin C*	*Tenascin C* for	5’-ACGGCTACCACAGAAGCTG-3’
	Tenascin C rev	5’-ATGGCTGTTGTTGCTATGGCA-3’
mouse *Endoglin 1*	*Endoglin 1 for*	5’-AGCTGGTCAGCCAGAAGAGT-3’
	Endoglin 1 rev	5’-GCCCTCGATCCAGGTGATCT-3’
mouse *Dermatopontin*	*Dermatopontin for*	5’-TGGATGGGTGAATCTTAACCGC-3’
	Dermatopontin rev	5’-TCAGAGCCTTCCTTCTTGCTA-3’
mouse *Col3a1*	*Col3a1 for*	5’-CTGTAACATGGAAACTGGGGAAA-3’
	Col3a1 rev	5’-CCATAGCTGAACTGAAAACCACC-3’
mouse *Biglycan*	*Biglycan for*	5’-AGACAAACCGACAGCCTGACAAC-3’
	Biglycan rev	5’-GCCAGCAGCAAGGTGAGTAGC-3’
mouse *SPARC*	*SPARC for*	5’-CCACACGTTTCTTTGAGACC-3’
	SPARC rev	5’-GATGTCCTGCTCCTTGATGC-3’
mouse *GAPDH*	*GAPDH* for	5’- AGGTCGGTGTGAACGGATTTG-3’
	GAPDH rev	5’- TGTAGACCATGTAGTTGAGGTCA-3’

Supplementary Table 3. List of 93 genes named “*Postn* linked genes”.

**6 Genes from intersection of PCd3>PCd0 with Postn linked genes and with PCd3>BMSCd3**	
Postn	periostin, osteoblast specific factor
Aspn	asporin
Col3a1	collagen, type III, alpha 1
Dcn	decorin
Egln1	EGL nine homolog 1 (C, elegans)
Lum	lumican
**16 Genes from intersection of PCd3>BMSCd3 with Postn linked genes**	
Bmpr1a	bone morphogenetic protein receptor, type 1A
Col12a1	collagen, type XII, alpha 1
Col1a1	collagen, type I, alpha 1
Col1a2	collagen, type I, alpha 2
Col5a1	collagen, type V, alpha 1
Col5a2	collagen, type V, alpha 2
Col6a3	collagen, type VI, alpha 3
Dpt	dermatopontin
Egfr	epidermal growth factor receptor
Fbn1	fibrillin 1
Mmp12	matrix metallopeptidase 12
Mmp2	matrix metallopeptidase 2
Mmp3	matrix metallopeptidase 3
Sparcl1	Sparc like 1
Tbx18	T-box18
Thbs2	thrombospondin 2
**9 Genes from intersection of PCd3>PCd0 with Postn linked genes**	
Bcl2l11	BCL2-like 1
Erbb3	V-erb-b2 erythroblastic leukemia viral oncogene homolog 3 (avian)
Grb2	growth factor receptor bound protein 2
Gtf2f2	general transcription factor IIF, polypeptide 2
Itgb2	integrin beta 2
Itgb5	integrin beta 5
Lgals3	lectin, galactose binding, soluble 3
Runx3	Runt Related Transcription Factor 3
Tgfb1	transforming growth factor, beta 1
**62 other Postn linked genes**	
Akt1	thymoma viral proto-oncogene 1
Bcar1	breast cancer anti-estrogen resistance 1
Bcl2l1	BCL2-like 1
Bgn	biglycan
Bmp1	bone morphogenetic protein 1
Bmp2	bone morphogenetic protein 2
Bmp4	bone morphogenetic protein 4
Cbl	Casitas B-lineage lymphoma
Cdh1	cadherin 1
Cdh11	cadherin 11
Cdx1	caudal type homeobox 1
Clca3	chloride channel calcium activated 3
Col14a1	collagen, type XIV, alpha 1
Col2a1	collagen, type II, alpha 1
Col4a1	collagen, type IV, alpha 1
Col6a1	collagen, type VI, alpha 1
Col6a2	collagen, type VI, alpha 2
Ctnnb1	catenin (cadherin associated protein), beta 1

Ctnnd1	catenin (cadherin associated protein), beta 11
Egln2	EGL nine homolog 2 (C, elegans)
Erbb2	V-erb-b2 erythroblastic leukemia viral oncogene homolog 2 (avian)
Erbb4	V-erb-b2 erythroblastic leukemia viral oncogene homolog 4 (avian)
Fkbp4	FK506 binding protein 4
Fkbp5	FK506 binding protein 5
Fn1	fibronectin 1
Foxo1	forkhead box O1
Foxo3	forkhead box O3
Itga3	integrin alpha 3
Itgam	integrin alpha M
Itgax	integrin alpha X
Itgb1	integrin beta 1
Itgb3	integrin beta 3
Itgb6	integrin beta 6
Kdr	kinase insert domain protein receptor
Mmp13	matrix metallopeptidase 13
Mmp1b	matrix metallopeptidase 1b
Mtor	mechanistic target of rapamycin (serine/threonine kinase)
Ndufs2	NADH dehydrogenase (ubiquinone) Fe-S protein 2
Ndufs3	NADH dehydrogenase (ubiquinone) Fe-S protein 3
Ndufs7	NADH dehydrogenase (ubiquinone) Fe-S protein 7
Ndufv1	NADH dehydrogenase (ubiquinone) flavoprotein 1
Ndufv2	NADH dehydrogenase (ubiquinone) flavoprotein 2
Nos3	nitric oxide synthase 3, endothelial cell
Notch1	Notch gene homolog 1 (Drosophila)
Nppa	natriuretic peptide type A
Ogn	osteoglycin
Pdgfrab	platelet derived growth factor receptor, beta polypeptide
Ptk2	PTK2 protein tyrosine kinase 2
Pxn	paxillin
Rbpj	recombination signal binding protein for immunoglobulin kappa J region
Serpinb2	serine (or cysteine) peptidase inhibitor, clade B, member 2
Rictor	RPTOR Independent Companion Of MTOR Complex 2
Runx2	Runt Related Transcription Factor 2
Shc1	src homology 2 domain-containing transforming protein C1
Snai2	snail homolog 2 (Drosophila)
Sost	sclerostin
Sparc	SPARC
Tgfb2	transforming growth factor, beta 2
Tnc	tenascin C
Twist1	twist homolog 1 (Drosophila)
Vegfa	vascular endothelial growth factor A
Vegfc	vascular endothelial growth factor C
